# Text mining method to unravel long COVID’s clinical condition in hospitalized patients

**DOI:** 10.1038/s41419-024-07043-4

**Published:** 2024-09-13

**Authors:** Pilar Tavares Veras Florentino, Vinícius de Oliveira Araújo, Henrique Zatti, Caio Vinícius Luis, Célia Regina Santos Cavalcanti, Matheus Henrique Citibaldi de Oliveira, Anderson Henrique França Figueredo Leão, Juracy Bertoldo Junior, George G. Caique Barbosa, Ernesto Ravera, Alberto Cebukin, Renata Bernardes David, Danilo Batista Vieira de Melo, Tales Mota Machado, Nancy C. J. Bellei, Viviane Boaventura, Manoel Barral-Netto, Soraya S. Smaili

**Affiliations:** 1 Laboratório de Medicina e Saúde Pública de Precisão (MeSP2), Instituto Gonçalo Moniz, Fundação Oswaldo Cruz, Salvador, Brazil; 2 Centro de Integração de Dados e Conhecimentos para a Saúde (CIDACS), Instituto Gonçalo Moniz, Fundação Oswaldo Cruz, Salvador, Brazil; 3 Faculdade de Medicina da Bahia, Universidade Federal da Bahia, Salvador, Brazil; 4 Departamento de Farmacologia, Escola Paulista de Medicina, Universidade Federal de São Paulo, São Paulo, Brazil; 5 Faculdade de Medicina, Universidade de São Paulo, São Paulo, Brazil; 6 Diretoria de Tecnologia da Informação, Universidade Federal de Ouro Preto, Ouro Preto, Brazil; 7 Disciplina de Moléstias Infecciosas, Escola Paulista de Medicina, Universidade Federal de São Paulo, São Paulo, Brazil

**Keywords:** Epidemiology, Viral infection

## Abstract

Long COVID is characterized by persistent that extends symptoms beyond established timeframes. Its varied presentation across different populations and healthcare systems poses significant challenges in understanding its clinical manifestations and implications. In this study, we present a novel application of text mining technique to automatically extract unstructured data from a long COVID survey conducted at a prominent university hospital in São Paulo, Brazil. Our phonetic text clustering (PTC) method enables the exploration of unstructured Electronic Healthcare Records (EHR) data to unify different written forms of similar terms into a single phonemic representation. We used n-gram text analysis to detect compound words and negated terms in Portuguese-BR, focusing on medical conditions and symptoms related to long COVID. By leveraging text mining, we aim to contribute to a deeper understanding of this chronic condition and its implications for healthcare systems globally. The model developed in this study has the potential for scalability and applicability in other healthcare settings, thereby supporting broader research efforts and informing clinical decision-making for long COVID patients.

## Introduction

Advances in emerging technologies such as artificial intelligence (AI) and machine learning (ML) hold promise for the development of healthcare transformation in prediction, contact tracing, screening, diagnosis and treatment, significantly improving medical practice [1–6]. One potential utility of AI is to assist in the extraction of information from electronic medical records. Natural language processing (NLP) is a subfield of AI that enables computers to learn from unstructured medical records and adapt to new language patterns over time, which can be useful for administrative and research purposes. Unlike NLP, which seeks to comprehend the overall meaning of text, text mining focus on addressing a particular problem in a specific domain determined in advance, potentially employing some NLP techniques in the process [7]. Although efforts have been made to use text mining to extract information from medical records in the English language, studies in languages other than English are still emerging [8, 9] and are urgently needed for specific systems.

Historically, clinically relevant information from electronic health records (EHRs) has been extracted via manual review by clinical experts, resulting in scalability and cost [10]. This is particularly evident for chronic diseases, where clinical notes are more common than structured medical records data [11]. These unstructured data provide a great opportunity to test the performance of text mining in automatically extracting clinically meaningful information, which may be useful for research and administrative purpose [10].

Long COVID is a chronic disease characterized by persistence of symptoms for more than 1 month (according to Central of Disease Control—CDC) or more than 3 months (according to World Health Organization—WHO) that still lacks a definitive clinical characterization. New tools that help perform a meticulous analysis of vast amounts of unstructured data from EHR can uncover patterns, symptoms, and outcomes that might otherwise elude traditional research methods enabling a deeper comprehension of long COVID. Recent descriptions of long COVID are based on studies conducted and compiled in 2021 and more detailed studies conducted in 2022 with the aim of reaching a consensus [12–14]. Based on these studies, many works have sought to highlight what actually occurs after SARS-CoV-2 infection. It remains unclear why the virus causes so many different symptoms affecting a variety of systems and what defines their frequency and prevalence. Thus, understanding what happens after COVID-19 and identifying which sequelae correspond to the postacute disease are increasingly important. Several symptoms can persist for many months or years, in addition to elevated risks of complications and death [15–17]. However, there are still may doubts and inconsistencies related to long COVID, especially regarding patients who were hospitalized, as well as the correlation between the length of hospitalization and the severity of the disease. Therefore, it is necessary to investigate and follow patients who were hospitalized with COVID-19 in different hospitals and to clearly identify the risk factors that require attention after COVID-19 and how these factors affect patients’ lives after the disease.

In this study, we sought to classify and automatically extract data from a long Covid survey from a large hospital in the city of São Paulo, one of the Brazilian cities most affected by the pandemic. We analyzed the EHR and created a model that can be applied in other hospitals.

## Materials and methods

### Study design and data source

This is a cross sectional study that uses a national database for training a model of text mining and EHR from a referral hospital for testing the model. The training dataset provided the tokens for text mining. We then performed text mining on the testing dataset and compared the results with those of manual human classification.

The training dataset was built from the information system for severe acute respiratory illness (SIVEP-Gripe), in which all COVID-19 hospital admissions and deaths in Brazil were registered by federal law. The SIVEP-Gripe is the national registration database for severe acute respiratory syndrome (SARS) in Brazil including COVID-19 data, and all COVID-19 hospitalizations and deaths. SARS is defined as an individual who presents with dyspnea/respiratory discomfort, persistent pressure or pain in the chest, oxygen saturation less than 95% without oxygen, or cyanosis of the lips or face (https://www.gov.br/saude/pt-br/assuntos/coronavirus/artigos/definicao-e-casos-suspeitos). This database has been widely used as a source for other epidemiological studies [18–21]. In the present work, the SIVEP-Gripe was used to create a token dictionary for unstructured text from clinical questionnaires. The dataset is publicly available at https://opendatasus.saude.gov.br/group/dados-sobre-srag.

The testing dataset was built from the EHR of patients who were hospitalized for COVID-19 at the Hospital São Paulo, the University Hospital (UH) of the Federal University of São Paulo (Unifesp), from March 2020 to June 2022 and were followed after discharge at a Post-COVID-19 Disease Unit (PCDU). The PCDU is a multidisciplinary unit where health professionals assist patients and administer a questionnaire to gather information on any prolonged signs or symptoms after the acute phase of COVID-19. This questionnaire includes information on acute COVID-19, evolution of the infection, medical history, and post-acute phase signs and symptoms. These data were also linked to demographic information and SARS-CoV-2 PCR results from patients.

Hospital São Paulo uses multiple information systems, resulting in the dispersion of relevant information across different databases. As an initial search strategy to extract data, terminologies related to the infectious disease COVID-19 were applied to the Clinical Notes Database (MongoDB) for the period from March 1, 2020 to September 30, 2022. A total of eight clinical encounter forms were preselected from this data structure, and the clinical records of 16,017 patients were collected. The form data were extracted and grouped into eight JSON (JavaScript object notation) files that were converted to the Tidy Data format and saved as CSV (comma separated values) text files. Following the analysis of the files by the technical-scientific team, the clinical encounter form “Post-COVID Care (Pneumo)”, containing records of 440 patients, was chosen for the analysis of demographic and hospital historical data. The second database of interest was the general patient record, a relational database (Oracle) that was queried using the Standard Query Language (SQL). We extracted data on emergency room visits, outpatient consultations, appointments, exam results, hospitalizations, and surgeries. The extracted data from this database were stored in *.XLSX (Microsoft Excel 2007) file format. The extracted medical conditions and patient symptoms were validated with the assistance of ambulatory pneumology and infectiology at the UH.

### Clinical data collection

The SIVEP-Gripe dataset (training dataset), which contains ≥2.6 million entries and information on medical conditions and symptoms in the form of unstructured text, was used for the training dataset. Comorbidity and symptom information was extracted from this system via a phoneme approach using the metaphonept-br library (https://github.com/carlosjordao/metaphone-ptbr). All data processing was run in Python (Version: 3.10) using Jupyter Notebooks.

Before beginning the text analysis, it was necessary to normalize and clean the text strings. To this end, we utilized regular expressions to clean special characters and to make a diverse set of separators between words uniform to a whitespace. The phonetic text clustering (PTC) method groups terms together according to their phonetics, effectively consolidating variations of similar terms into a single phonemic representation and using n-gram text analysis to detect compound words [22]. This method not only captured and grouped terms but also allowed the accommodation of synonyms, abbreviations, typographical errors, and the different conjunctions found in Brazilian Portuguese.

To ensure the accuracy of these results, a dictionary of similar terms was carefully curated by five specialists in internal medicine, infectology, pharmacology, pathology, otorhinolaryngology and public health and three medical students. This step prevented the grouping of different terms into the same phoneme. This curation process was crucial in enabling the use of the dictionary to identify medical conditions from unstructured text from different and more complex contexts. Scripts from methodology employed are found in https://github.com/CampusVirtualFiocruz/Text-Mining-Clinical-Data-UNIFESP.

### PTC validation with long COVID questionnaires

To validate the PTC method, the clinical information of patients collected from the long COVID questionnaire was organized into structured and unstructured data (Testing dataset). The structured data consisted of yes–no and multiple-choice answers, as well as numerical variables. The unstructured data comprised textual patient reports, including records of symptoms, clinical signs, laboratory tests, previous medical conditions, and lifestyle habits, such as smoking.

Subsequently, an automated approach to process the unstructured variables from the long COVID questionnaires was applied. We searched for all previously defined terms in the curated dictionary and focused on the most frequent comorbidities associated with COVID-19, which included obesity, hypertension, diabetes mellitus, chronic obstructive pulmonary disease (COPD), asthma, hypothyroidism, and hyperthyroidism. Information on patients’ smoking history was also collected to classify patients as smokers or former smokers. Medical records that did not include information on comorbidities or smoking history were classified as having no comorbidities or having a non-smoking history.

In addition, information on long COVID symptoms (cough, fatigue, headache and myalgia) (https://www.cdc.gov/coronavirus/2019-ncov/long-term-effects/index.html) was collected. For symptoms, the focus was on terms in the questionnaires that described patient information in the present, excluding the text referring to symptoms reported in the past (acute phase).

**Fig. 1:**
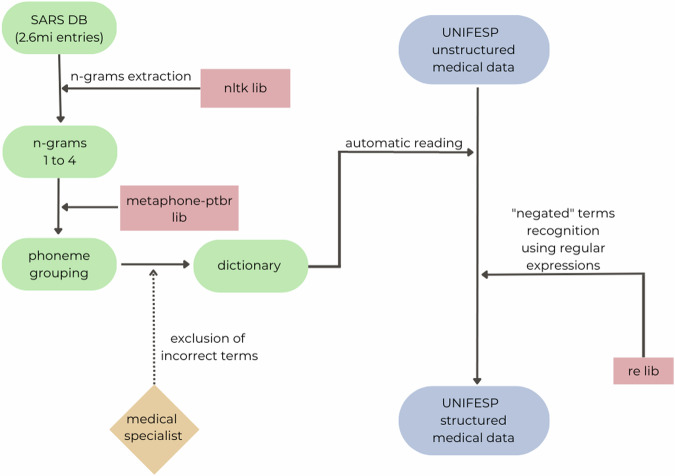
Diagram detailing the method developed during the present study and the dataset extracted for further analyses. The figure shows the process of extracting and processing clinical data using phonetic text clustering (PTC) from the SARS database (2.6 million entries) and UNIFESP unstructured medical data. n-grams (1–4) are extracted using the NLTK library, grouped by phonemes with the metaphone-ptbr library, and used to create a dictionary. A medical specialist validates the dictionary by excluding incorrect terms. UNIFESP unstructured medical data are automatically read, with negated terms recognized using regular expressions (re lib), and integrated into the UNIFESP structured medical data. This process combines automated text processing with manual validation to improve data accuracy and completeness for analysis.

Importantly, the medical condition terms detected in the unstructured text of the long COVID dataset could be in the context of a negative report, i.e., the patient confirming or denying the medical condition. To address this issue, we assessed negative operators such as “deny” (*nega*, in Brazilian Portuguese) that appeared before the comorbidity of interest and until the following sentence with regular expressions (https://docs.python.org/3/library/re.html). This allowed us to capture instances where patients denied having the specified condition. Figure 1 shows a diagram detailing the method developed in this study using the extracted dataset. Scripts from methodology employed can be found in https://github.com/CampusVirtualFiocruz/Text-Mining-Clinical-Data-UNIFESP.

### Long COVID study population

The study population included adults (≥18 years) who were hospitalized due to acute COVID-19 at the Hospital São Paulo and discharged. The data were collected between March 2020 and June 2022. We excluded individuals who (1) had no SARS-CoV-2 PCR result or had a negative result; (2) did not have a recorded date of first symptoms; (3) completed questionnaires less than 30 days after the first occurrence of symptoms; (4) were hospitalized for more than 120 days; (5) did not have records of COVID-19 evolution during the acute phase; (6) had date inconsistencies; (7) had encounters after the first questionnaire; or (8) had no severity classification in the acute phase.

Demographic variables from the long COVID dataset, such as sex, age (stratified into 18–39, 40–59, 60–79, and ≥80 years), and race (divided into white, black, mixed-brown, Asian, and Indigenous), were evaluated. Due to the small sample size, Asian and Indigenous individuals were combined for the analysis. Additionally, other variables, such as medical history, length of hospital stay (stratified into 0–14, 15–30, 31–60, and ≥60 days), and severity of the acute phase of COVID-19, were included. The severity categories were defined as moderate (non-ICU ward), severe (intensive care unit; ICU), or critical (ICU with mechanical ventilation).

### Statistical analysis

To validate the accuracy of the automated approach, we compared the automated results with manually searched and labeled clinical data. The manual labeling was performed by six of the authors with clinical training, and each record was individually labeled three times. We conducted Pearson’s chi-square test to compare the automated and manual term counts to assess the accuracy of the text mining. Then, we performed a descriptive analysis of the long COVID findings to validate our findings against those of previous studies on the topic. Also, we performed a generalized linear model (GLM) to assess the relationship between the demographic characteristics and the presence of post-COVID-19 symptoms.

### Ethical approval

The Brazilian National Commission in Research Ethics approved the research protocol (CONEP approval number 4.921.308 and CAAE registration no. 58619822.6.1001.5505).

## Results

### Automated labeling of the training dataset

First, we investigated the records from the SIVEP-Gripe. A total of 2,490,196 SARS records of patients admitted to hospitals between December 31, 2019, and March 27, 2023 were collected. All records were then analyzed as input for the PTC tokenization of medical conditions and symptoms, which were used to create the dictionary that was used to structure the data and to create the database for long COVID. Overall, 635,921 (25.5%) records reported one or more medical conditions, and 849,976 (34.1%) reported one or more SARS-related symptoms in the unstructured text field (Fig. S1 and Table 1). From the unstructured clinical data, a dictionary collecting synonyms, misspelled and derivative words into a unique term (Table S1) was produced. Based on this dictionary, 20 of the most frequent medical conditions and 10 of the most frequent symptoms (Table 1) were captured for further analyses.

**Table 1:** Gain of information from clinical data extracted by phonetic text clustering (PTC) in hospitalized patients with severe acute respiratory syndrome (SARS).

Terms	Category	Before automated reading	After automated reading	Overlap	Gain	Binary variable
Hypertension	Medical condition	0	303,109	0	303,109	No
Smoker	Medical condition	0	61,110	0	61,110	No
Hypothyroidism	Medical condition	0	39,550	0	39,550	No
Cardiopathy	Medical condition	797,995	831,684	21,244	12,445	Yes
COPD	Medical condition	0	30,387	0	30,387	No
Former smoker	Medical condition	0	23,189	0	23,189	No
Alzheimer	Medical condition	0	16,323	0	16,323	No
Alcoholism	Medical condition	0	16,105	0	16,105	No
Obesity	Medical condition	180,118	194,958	1068	13,772	Yes
Depression	Medical condition	0	14,348	0	14,348	No
Breast cancer	Medical condition	0	7146	0	7146	No
Epilepsy	Medical condition	0	5976	0	5976	No
Parkinson	Medical condition	0	5281	0	5281	No
Schizophrenia	Medical condition	0	4340	0	4340	No
Lymphoma	Medical condition	0	2915	0	2915	No
Rheumatoid arthritis	Medical condition	0	2628	0	2628	No
Hyperthyroidism	Medical condition	0	1819	0	1819	No
Rhinitis	Medical condition	0	1314	0	1314	No
Diabetes mellitus	Medical condition	540,840	541,357	140	377	Yes
Chronic kidney disease	Medical condition	92,107	92,130	6	17	Yes
Headache	Symptoms	0	215,225	0	215,225	No
Myalgia	Symptoms	0	213,035	0	213,035	No
Asthenia	Symptoms	0	124,086	0	124,086	No
Runny nose	Symptoms	0	122,766	0	122,766	No
Lack of appetite	Symptoms	0	69,990	0	69,990	No
Nausea	Symptoms	0	55,461	0	55,461	No
Loss of smell	Symptoms	175,934	186,958	635	11,024	Yes
Loss of taste	Symptoms	179,480	184,141	370	4661	Yes
Fatigue	Symptoms	567,290	590,766	3182	20,294	Yes
Cough	Symptoms	1,796,926	1,797,121	1317	195	Yes
		4,330,690	5,755,218	27,962	1,398,888	

The table compares the identification counts of various medical conditions and symptoms before and after implementing PTC. It includes the terms assessed (Terms), their category, counts before and after automated reading, overlap of counts from structured and unstructured information, gain in identification with PTC, and a binary variable indicating whether this condition already appeared as structured text in the dataset.

**Fig. 2:**
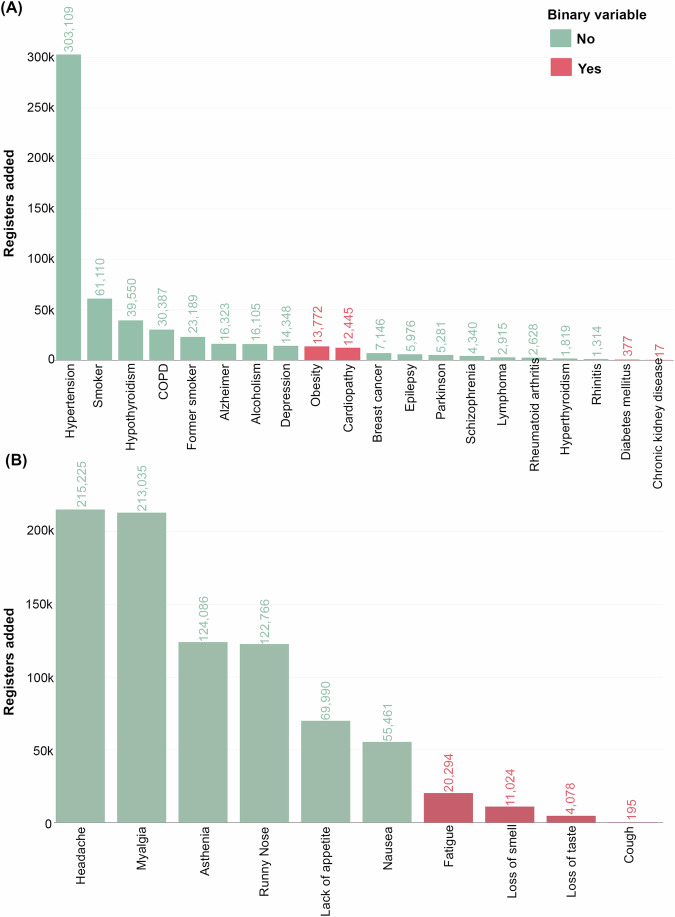
Gain of information from clinical data extracted by the phonetic text clustering (PTC) method in hospitalized patients with severe acute respiratory syndrome (SARS) in Brazil. The figure shows the most frequent terms captured using PTC. **A** displays the most frequent terms related to medical conditions, highlighting conditions such as hypertension, smoker status, and hypothyroidism, among others. **B** shows the most frequent terms associated with symptoms, including headache, myalgia, and asthenia. The bar colors indicate whether the terms appeared in the structured data before the application of PTC (red for “Yes”, green for “No”).

SARS patient records were stratified by medical conditions and symptoms in a “yes/no” format, such as diabetes mellitus, obesity, cardiopathy, loss of smell, loss of taste, fatigue and cough. The results showed that 22,458 of the terms containing medical conditions captured from the unstructured text overlapped with at least one of the binary comorbidities with a “yes” response in the questionnaire. In addition, 1418 terms overlapped with at least one of the binary symptom variables with a “yes” response. Thus, to evaluate the gain of information, records with overlapping medical conditions or symptoms were excluded. The terms that were not included in the binary variables from the questionnaire appeared more frequently in the unstructured text annotation (Fig. 2).

Among medical conditions, the most frequent term captured by the automated reading was “hypertension”, present in 303,109 entries, representing 11.3% of the total database (Fig. 2A), followed by “smoker” in 61,110 entries, representing 2.3%; “hypothyroidism” in 39,550 entries, representing 1.5%; and “COPD” in 30,387 entries, representing 1.1%. Additionally, “smoking” was found in 61,110 entries (2.3%). Among symptoms, the most frequent terms were “headache”, present in 215,225 entries, representing 8.0% of the total database; “myalgia”, present in 213,035 entries, representing 7.9%; “asthenia”, present in 124,086 entries, representing 4.6%; and “runny nose”, present in 122,766 entries, representing 4.5% (Fig. 2B).

### Validating text mining on EHRs

Data from patients who were admitted with COVID-19 at the Hospital São Paulo, stayed in the hospital for more than 30 days, and were followed at the PCDU after discharge were evaluated.

**Fig. 3:**
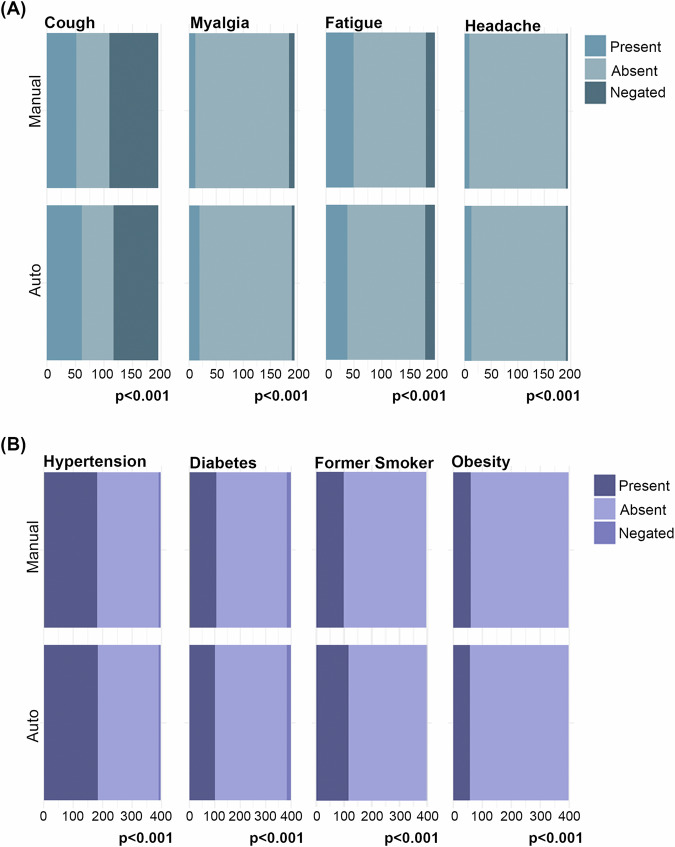
Comparison between the manual and automated methods of the most frequently reported terms in unstructured text in the population studied. This figure compares the performance of manual versus automated methods in identifying the most frequently reported terms in unstructured text for **A** symptoms such as cough, myalgia, fatigue, and headache, and **B** medical conditions including hypertension, diabetes, former smoker status, and obesity. The bar plots indicate the counts of terms identified as present, absent, or negated, with significant differences between methods (*p* < 0.001). The upper bars represent the manual method, and the lower bars represent the automated method.

To validate the PTC method on these data obtained from Hospital São Paulo, 398 post-COVID patient questionnaires collected from the PCDU (Fig. S2) were cross-checked. The dictionary derived from records of SARS-hospitalized patients was applied. Medical conditions and symptoms from these post-COVID-19 patients were extracted and studied by using an automated method. The results obtained were compared with those obtained through manual searches conducted by specialists, which showed a high degree of similarity in present, absent and negated terms. According to this method, the similarity ranged from 93% to 99% for medical condition terms and from 87% to 95% for symptom terms (Table S2). The statistical significance of these findings is reflected in the *p* values for all terms, which were less than 0.01 (Fig. 3 and Table S2).

The study population was divided into individuals who reported no symptoms (29.1%) and those with at least one symptom 30 days after COVID-19 onset (70.9%) (Table 2). Data revealed that 24% of patients with three or more medical conditions showed post-COVID-19 symptoms after 30 days of discharge from the hospital (24% with symptoms against 17% without symptoms).

**Table 2:** Description of the population with long COVID (demographic) with and without comorbidities.

Characteristic	Without symptoms *n* = 116	With symptoms *n* = 282
Gender		
F	49 (42%)	123 (44%)
M	67 (58%)	159 (56%)
Ethnicity		
Black	11 (9.5%)	35 (12%)
White	64 (55%)	170 (60%)
Mixed-Brown	39 (34%)	68 (24%)
Asian/Indigenous	2 (1.7%)	9 (3.2%)
Age range		
18–39	13 (11%)	35 (12%)
40–59	60 (52%)	110 (39%)
60–79	37 (32%)	121 (43%)
80+	6 (5.2%)	16 (5.7%)
Severity		
Moderate	63 (54%)	122 (43%)
Severe	27 (23%)	89 (32%)
Critical	26 (22%)	71 (25%)
Days after 1st symptoms in acute phase		
30–90 days	87 (75%)	202 (72%)
90–120 days	18 (16%)	45 (16%)
120–180 days	10 (8.6%)	22 (7.8%)
180–240 days	0 (0%)	6 (2.1%)
240 days	1 (0.9%)	7 (2.5%)
Days of hospitalization		
0–14 days	72 (62%)	143 (51%)
15–30 days	27 (23%)	87 (31%)
30–60 days	12 (10%)	37 (13%)
60 days	5 (4.3%)	15 (5.3%)
Number of medical conditions		
No medical condition	36 (31%)	66 (23%)
1 medical condition	32 (28%)	76 (27%)
2 medical conditions	28 (24%)	72 (26%)
3+ medical conditions	20 (17%)	68 (24%)

This table provides a demographic overview of the population studied, categorizing individuals with long COVID into those with and without comorbidities. The table includes variables such as age, gender, ethnicity, and other relevant demographic factors.

The demographic data revealed that both groups had a similar gender distribution, with slightly more males (58% without symptoms, 56% with symptoms). Ethnicity distribution indicated that self-declared white individuals were the majority in both groups (55% without symptoms, 60% with symptoms), followed by mixed brown (34% without symptoms, 24% with symptoms). Furthermore, the symptomatic group had a higher percentage of older individuals (43% aged 60–79) compared to the asymptomatic group (32% aged 60–79). Although a slight difference was observed between both groups. The results from generalized linear model (GLM) to assess the relationship between the demographic characteristics and the presence of post-COVID-19 symptoms showed no statistically significant differences (Table S3).

**Fig. 4:**
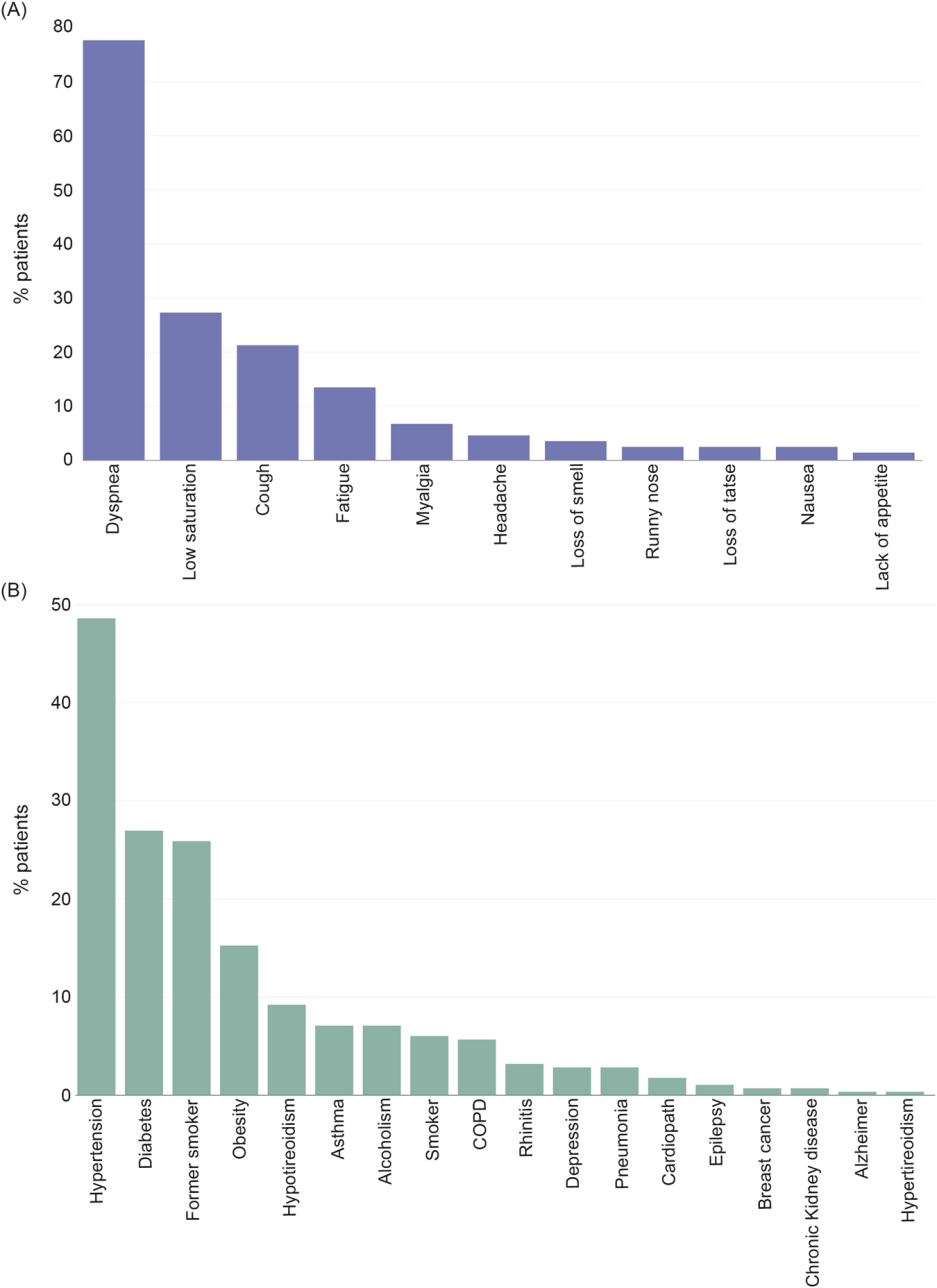
Based on the methods developed and validated, the most prevalent symptoms and medical conditions related to long COVID were investigated and analyzed in the study population. The most common symptoms and medical conditions reported by patients in the study are represented in the figure. **A** shows the percentage of patients with symptoms and **B** displays the percentage of patients with medical conditions. The data were collected through questionnaires and reveal the prevalence of these conditions in the study population.

For patients who presented with at least one symptom, the most prevalent symptom was dyspnea (77.7%), followed by cough (21.3%) and fatigue (13.5%). Low oxygen saturation (below 27.3%) was the most common continuous variable reported. In terms of lifestyle, 25.9% were former smokers. A total of 48.6% of the population with symptoms after 30 days had hypertension, 26.9% had diabetes, and 15.2% had obesity (Fig. 4).

## Discussion

Three different developments resulted from this study. First, we built a text mining workflow that was able to extract structured medical information from clinical notes in Brazilian Portuguese. Second, this method, in conjunction with the validated text tokens, could be used as a platform for future analyses of long COVID in hospitals that use different systems. Finally, the method was applied back to the training dataset (SIVEP-Gripe), enriching the national database and resulting in more detailed clinical characterizations of SARS in Brazil in the last decade.

The method developed for text mining of clinical data was based on grouping synonyms by phoneme. Our method was able to extract clinical information that was not available previously as variables, with a total informational gain of 32.30% for the 30 categories of comorbidities and symptoms from the records of hospitalized SARS patients. Furthermore, we validated our method against human labeling using electronic records from patients who returned to the post-COVID-19 unit after being discharged for 30 days, which allowed us to describe the clinical findings related to long COVID in those patients.

The initial difficulty was structuring a database from a set of unstructured data that would allow subsequent analysis of a disease such as COVID-19 and post acute symptoms, characterized as long COVID. The benchmarks used were previous studies on COVID-19 and vaccine effectiveness using national health system datasets, from which cohorts for studies on the effectiveness of different vaccines administered in Brazil were formed, and gathered at the national databases [20, 23]. Thus, it was possible to enrich the same dataset and cross-check the informational gain using data from patients who were admitted to the UH and who, after discharge, were followed up at the PCDU due to various symptoms.

The method developed in this study exhibited robust performance and was subsequently used to investigate the effects of long COVID in patients who were admitted to the UH and were followed for several months after being discharged. Phonemic representation has been used previously to cluster variations in writing and represent these clusters as an n-gram [24, 25], but this is, for the best of our knowledge, the first time that it has been used for clinical notes in Brazilian Portuguese [22]. This plot captured groups of variations in terms, such as close synonyms, abbreviations, and typographical errors typical of the language, which confirmed the validation and interpretability of the PTC method.

Importantly, the construction of this method allowed for a more accurate analysis of symptoms in patients followed by the PCDU of Hospital São Paulo, which showed that the majority of individuals presented dyspnea as a prevalent symptom, often accompanied by low oxygen saturation. These data are in accordance with other studies that used different methods, including the studies that reported low oxygen saturation during physical exercise [26, 27]. Since dyspnea is one of the most frequent and well-documented symptoms of long COVID [28], it is notable that, besides its detection, our study and method provided further information concerning low oxygen saturation. In addition, other symptoms, such as fatigue and muscle pain, were detected and had been described by other authors [29], corroborating the quality of the new method to extract symptoms from non-structured data.

Importantly, the curation and constant maintenance of the dictionary will be continued, and we will update it with new information and terms used by other health services. Thus, new qualifiers of clinical conditions, such as different degrees of dyspnea and the evolution of these clinical conditions over time, which may encompass periods of improvement and worsening, will be included in the dictionary. In addition, creating specific platforms to characterize and identify a little-known and difficult-to-diagnose condition, such as long COVID, represents an important advance for data modeling and decision-making after the occurrence of COVID-19. The tool created from the methods used in this study has characteristics that indicate the possibility of analyzing data in the language in which medical records are written, in addition to machine and human checking, which can overcome the lack of homogeneity in different records and allow more accurate results. These results are important, although it is necessary to emphasize that the risks of death and hospitalization remained statistically high in different phases of the pandemic, particularly in those who were hospitalized during the acute phase of SARS-CoV-2 infection and in countries such as Brazil [30], in which a high number of cases were reported. Therefore, these countries must also consider the substantial number of individuals with COVID-19 sequelae and provide health care to the population. Since there is also evidence of COVID-19 sequelae in individuals who were not hospitalized, it is crucial to emphasize the importance of treating those who were infected and prevent reinfections. Therefore, reducing the risk of long-term sequelae remains a need in terms of public health and health policies.

Finally, there are still many gaps and regional disparities in long COVID research. In particular, there are significant geographic gaps in the available research data, with an abundance of studies originating from Northern Hemisphere populations and a paucity of information regarding long COVID in low- and middle-income countries. There is a critical need for more focused research in these regions. Therefore, the use of text mining to evaluate non-structured EHRs provides a great opportunity to improve the knowledge of long COVID in areas with resource-limited settings.

The method and modeling presented in this work and the use of cohorts of data to predict and treat long COVID patients will be crucial, and more studies should be performed to not only increase knowledge but also develop the necessary care and rehabilitation methods in addition to the planning of the primary health care system. In this context, studies such as the present one should be expanded to help understand long COVID and predict its effects. These studies will allow the development of prevention and treatment that will lead to higher quality standards in population health even in the face of the a pandemic.

## Supplementary information

Supplementary Material

## Data Availability

Due to ethical and legal reasons, supporting data are not available.
