# Supplementary material for: Text mining method to unravel long COVID’s clinical condition in hospitalized patients
Source: Cell Death Dis. 2024 Sep 13;15(9):671. doi: 10.1038/s41419-024-07043-4 (PMC11399332; doi:10.1038/s41419-024-07043-4)
Supplement: Supplementary file 1 — Supplementary Material [file 41419_2024_7043_MOESM1_ESM.docx]

**SUPPLEMENTARY INFORMATION**

**Text Mining method to Unravel Long COVID's clinical condition in hospitalized patients**

Pilar Tavares Veras Florentino^1,2*^, Vinícius de Oliveira Araújo^2,3 *^, Henrique Zatti^2*^, Caio Vinícius Luis^4^, Célia Regina Santos Cavalcanti^4^, Matheus Henrique Citibaldi de Oliveira^4^, Anderson H. F. F. Leão^4^, Juracy Bertoldo Junior^2^, George G Caique Barbosa^1^, Ernesto Ravera^4^, Alberto Cebukin^4^, Renata David^4^, Danilo Batista Vieira de Melo^1^, Tales Mota Machado^5^, Nancy Bellei^4^, Viviane Boaventura^1,3^, Manoel Barral-Netto^1,3**,^ Soraya Smaili^4^**

Sumário

[Figure S1. Selection of records with text field information on symptoms and medical conditions from the training dataset (SIVEP-Gripe) to create a dictionary. 2](#_Toc171966197)

[Table S1. Dictionary of medical condition and symptom terms obtained from the records of hospitalized SARS patients. 2](#_Toc171966198)

[Figure S2. Selection of patients followed up by the post-COVID-19 disease unit (PCDU). 36](#_Toc171966199)

[Table S2. Comparison between manual and automated reading of medical condition and symptom terms. 37](#_Toc171966200)

[TABLE S3. Odds Ratio with 95% confidence interval from study population with and without long Covid reported symptoms. 37](#_Toc171966201)


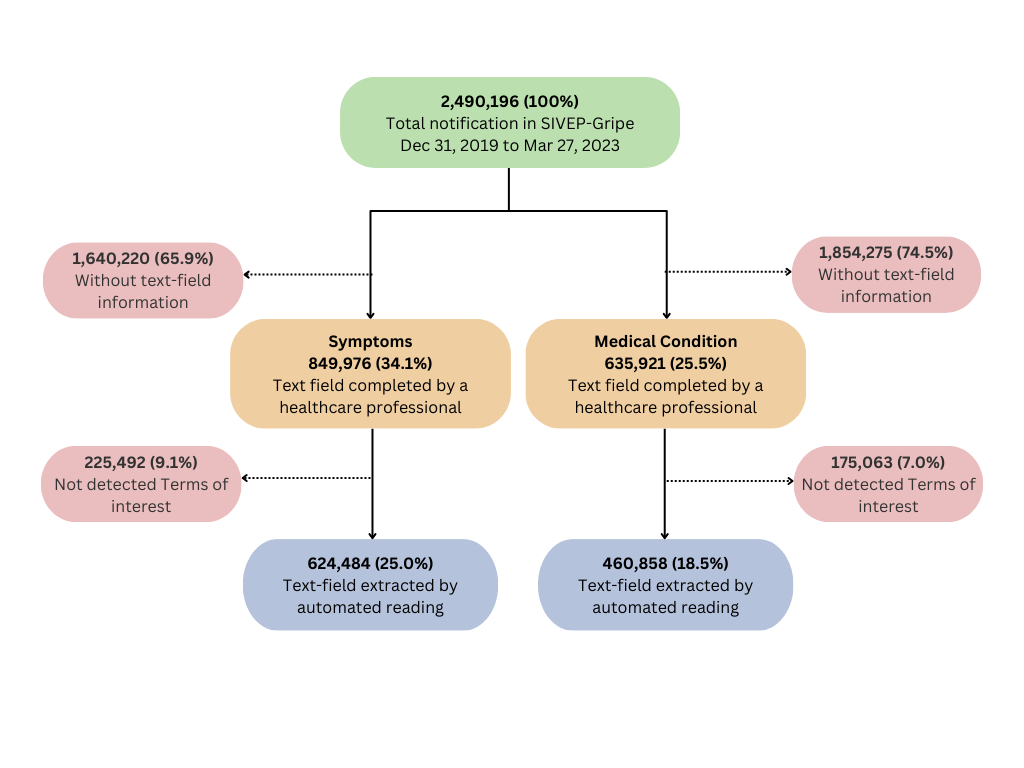


# **Figure S1.** Selection of records with text field information on symptoms and medical conditions from the training dataset (SIVEP-Gripe) to create a dictionary.

# **Table S1.** Dictionary of medical condition and symptom terms obtained from the records of hospitalized SARS patients.

| **Term (ENG)** | **Term (PT-BR)** | **Synonym** | **Category** | **Phoneme** | **Detected words** |
| --- | --- | --- | --- | --- | --- |
| Hypertension | hipertensao | has | Medical condition | AS | ['HAS', 'HAAS', 'HASS'] |
| Hypertension | hipertensao | hipertensao | Medical condition | IPRT | ['HIPERTENSAO', 'HIPERTENSO', 'HIPERTENSA', 'HIPERTENCAO', 'HIPERTESAO', 'HIPERT', 'HIPERTENSIVO', 'HIPERETENSAO', 'HIPERTENSIVA', 'HIPERTENSSAO', 'HIPERTENS', 'HIPERETNSAO', 'HIIPERTENSA', 'HIIPERTENSAO', 'HIPERTENCA', 'HIPERATIVO', 'HIPERTEN', 'HIPERTNSAO', 'HIPRTENSAO', 'HIPERTANSAO', 'IPERTENSAO', 'HIPERTENAO', 'HIPERTECAO', 'HIPERTESO', 'HIPERTENCAOA', 'HIPERTENSAP', 'HIPERTE', 'HIPERTRENSAO', 'HIPERTNSO', 'HIPERTNSA', 'HIPERTTENSAO', 'HIPRETENSAO', 'HIPERTERSAO', 'HIPERTERNSAO', 'HIPERETENSA', 'HIPEERTENSAO', 'HIPOERTENSAO', 'HIPERTENSIVOS', 'HIPERTI', 'HIPERTERMIA', 'HIPERTENO', 'HIPERTPENSAO', 'HIPERATIVA', 'HIPERTRI', 'HIPERTIROI', 'IPERTENCAO', 'IPERTENCA', 'HIPERTENCO', 'HIPERTENCIA', 'HIPERTERNSO', 'HIPERTESA', 'HIPERTENSIV', 'HIPORTENSAO', 'HIPORTONIA', 'HIPERTESNAO', 'HIPERTESN', 'HIPERTERCAO', 'HIPERTENTENSAO', 'HIPERTENTENCAO', 'HIPERTENSINA', 'HIPERTENSI', 'HIPERTESSAO', 'HIPERTENC', 'HIPERTENAAO', 'HIPERTANCAO', 'HIPERTACAO', 'HIPERTNCAO', 'HIPERTNESAO', 'HIPERTRNSAO', 'HIUPERTENSAO', 'HIOPERTENSAO', 'IPERTENSO', 'HIPORITEO', 'HIEPRTENCAO', 'HIEPRTENSAO', 'HIEPERTENSO', 'HHIPERTENSO', 'HHIPERTENSAO', 'HHIPERTENSA', 'HIPERTENCAODEPRESSAO', 'HIPERTENCAOSRTERIAL', 'HIPERTENCE', 'HIPERTENCIVA', 'HIPERTENDO', 'HIPERTENASAO', 'HIPERTENIA', 'HIPERTENSAAO', 'HIPERTENSAI', 'HIPERTENSAL', 'HIPERTENSAOARTERIAL', 'HIPERTENBSAO', 'HIPERTAO', 'HIPERTEBSAO', 'HIPERTELORIRSMO', 'HIPERTEMIA', 'HIPERTEMSAO', 'HIPERTENSAOARTERIALSISTEMICA', 'HIPERTENSAOCOLESTEROL', 'HIPERTENSAODISTURBIO', 'HIPERTERNCAO', 'HIPERTES', 'HIPERTENSAOE', 'HIPERTESNSA', 'HIPERTENSSA', 'HIPERTENSAOM', 'HIPERTENSAON', 'HIPERTENSAOQ', 'HIPERTENSAS', 'HIPERTENSCAO', 'HIPERTENSE', 'HIPERTENSK', 'HIPERTENSOA', 'HIPERTENSODO', 'HIPERTENSOHAS', 'HIPERTENSOS', 'HIPERETENSO', 'HIOPERTENCAO', 'HIPIRTENSAO', 'HIPIRTENSO', 'HIPERTNS', 'HIPPERTENSAO', 'HIPRETENSO', 'HIPRTENSO', 'HIPRTENSSAO'] |
| Diabetes | diabetes | dm | Medical condition | DM | ['DM', 'DMII'] |
| Diabetes | diabetes | diabetes | Medical condition | DBTS | ['DIABETES', 'DIABETIS', 'DIABETTES', 'DOIABETES'] |
| Smoker | tabagismo | tabagismo | Medical condition | TBJS | ['TABAGISTA', 'TABAGISMO', 'TABAGIS', 'TABAGIST', 'TABGISTA', 'TABAGISM', 'TBAGISTA', 'TABAJISTA', 'TABAGISTAS', 'TABGISMO', 'TABAGIOSTA', 'TBGISTA', 'TABAJISMO', 'TABAGISTMO', 'TABAGISNO', 'TABAGISMOS', 'TABAGISTO', 'TBAGISMO', 'TABIGISTA', 'TABAGISMA', 'TABAGIASTA', 'TEBAJISTA', 'TABAGGISTA', 'TABAGISTATRANSTORNO', 'TABAGISMODPOC', 'TABGIST', 'TABAGISTATB', 'TABBAGISTA', 'TABAGISTI', 'TABAHGISMO', 'TABAGISTAFUMOU', 'TABAGISMMO', 'TABAGISTAA', 'TABAGISTAATIVO', 'TABAGISTAE', 'TABAGISTAEX', 'TAABAGISTA', 'TAABGISTA', 'TEBAGISTA', 'TOBAGISTA', 'TALBAGISTA', 'TIBAGIS', 'TABAJIST', 'TABAJISTATA', 'TABALGISTA', 'TABAGISTAARRITMIA', 'TABAGISTAETILISTA', 'TABAGISTAHA', 'TABAGISTAHIPERUCEMIA', 'TABAGISTAT', 'TABAGISTGA', 'TABAGISTIAM', 'TABAGISTQA', 'TABAGISTS', 'TABAGIUSTYA', 'TABOGISTA', 'TABBAGISMO', 'TABIGISMO', 'TAABAAGISTA', 'TAABAGISMO', 'TABAAGISTA', 'TABAGISMISMO', 'TABAGISMOA', 'TABAGISMOO', 'TABAGISMOP', 'TABAGISMOPO', 'TABAGISNMO', 'TABAGESTA', 'TABAGHISTA', 'TABAGIASMO'] |
| Smoker | tabagismo | fumante | Medical condition | FMNT | ['FUMANTE', 'FUMANT', 'FULMANTE', 'FUMNTE'] |
| Former smoker | ex tabagista | ex tabagista | Medical condition | EX TBJS | ['EX TABAGISTA', 'EX TABAGISMO', 'EX TABAGIST', 'EX TABAGIS', 'EX TABGISTA', 'EX TBAGISTA', 'EX TABAJISTA', 'EX TABAGISTAS', 'EX TBGISTA', 'EX TABAGISTATRANSTORNO', 'EX TABIGISTA', 'EX TABAJISMO', 'EX TABAGISTAFUMOU', 'EX TABAGISTAEX', 'EX TABAGISTAA', 'EX TABAGISM', 'EX TAABAGISTA', 'EX TBAGISMO', 'EX TABGISMO', 'EX TABAJISTATA', 'EX TEBAGISTA', 'EX TOBAGISTA', 'EX TABAGGISTA', 'EX TABAAGISTA', 'EX TABAGHISTA', 'EX TABAGIASTA', 'EX TABAGISTGA', 'EX TABAGISTS', 'EX TABAGISTIAM', 'EX TABAGISTI', 'EX TABAGISTAT', 'EX TABAGISTAHIPERUCEMIA', 'EX TABAGISTAE', 'EX TABAGISTAARRITMIA', 'EX TABAGISMOS', 'EX TABAGISMA'] |
| Hypothyroidism | hipotireoidismo | hipotireoidismo | Medical condition | IPTR | ['HIPOTIREOIDISMO', 'HIPOTIROIDISMO', 'HIPOTIREODISMO', 'HIPOTIREIODISMO', 'HIPOTIREOID', 'HIPOTEREOIDISMO', 'HIPOTIREOIDISM', 'HIPOTIREIDISMO', 'HIPOTIREOIDIS', 'HIPOTIREOI', 'HIPOTIREO', 'HIPOTIREOIDE', 'HIPOTIRIOIDISMO', 'HIPOTIRE', 'HIPOTIREOIDI', 'HIPOTIRIODISMO', 'HIPOTIROEDISMO', 'HIPOTEROIDISMO', 'HIPOTERIODISMO', 'HIPOTIREOISDISMO', 'HIPOTIREIOIDISMO', 'HIPOTIROID', 'HIPOTIROIDI', 'HIPOTIRODISMO', 'HIPOTIREOIDIMO', 'HIPOTIROIDIS', 'HIPOTOREOIDISMO', 'HIPOTEREIODISMO', 'HIPOTIROIDISM', 'HIPOTIRO', 'HIPOTIROI', 'HIPOTERIOIDISMO', 'HIPOTIROISMO', 'HIPOTEIREOIDISMO', 'HIPOTIREOIDEO', 'HIPOTEREODISMO', 'HIPOTIREOEDISMO', 'HIPETIREOIDISMO', 'HIPOTIREOIDSMO', 'HIPOTIEREOIDISMO', 'HIPOTIREODI', 'HIPOTIREOD', 'HIPOTIROISDISMO', 'HIPETIROIDISMO', 'HIPOTIREOIDEA', 'HIPOTIREOISIMO', 'HIPETIREODISMO', 'HIPOTIRIDISMO', 'HIPOTIEROIDISMO', 'HIPOTOROIDISMO', 'HIPOTIROEIDISMO', 'HIPOTIROIDIMO', 'HIPOTIREODIS', 'HIPOTIREDISMO', 'HIPOTIREOIDISMOS', 'HIPOTIROIDEISMO', 'HIPOTIREOIDEISMO', 'HIPOTIROIDISMOS', 'HIPOTREOIDISMO', 'HIPOTIREOIDIMSO', 'HIPOTIORIDISMO', 'HIPOTIREODISM', 'IPOTIREOIDISMO', 'HIPOITIREOIDISMO', 'HIPOTIOREOIDISMO', 'HIPOTIREOISMO', 'HIPOTIROIDEA', 'HIPOTIROIDSMO', 'HIPOTIRTEOIDISMO', 'HIPOTEIROIDISMO', 'HIPOTEREADISMO', 'HIOPOTIREOIDISMO', 'HIPOTIREOIDSIMO', 'HIPOITIROIDISMO', 'HIPOTEREOI', 'HIPITIREOIDISMO', 'HIPOTERODISMO', 'HIPOTROIDISMO', 'HIPOTIREADISMO', 'HIPOTIREOIDESMO', 'HIPOTIREOIDIMOS', 'HIPOTIROIDE', 'HIPOTIREOISISMO', 'HIPOTIRIOISDISMO', 'HIPOTIREIODIS', 'HIPOTIREODIMO', 'HIPOTIREI', 'HIPOTIROPIDISMO', 'HIPOTIROIRDISMO', 'HIPTIREOIDISMO', 'HIPPOTIREOIDISMO', 'HIPITIROIDISMO', 'HIPOTIREODE', 'HIPOTIREIODISM', 'HIPOTIREOIDEIA', 'HIPOTIREOIDDISMO', 'HIPOTIREOEIDISMO', 'HIPOTIREODISMIO', 'HIPOTIREIO', 'HIPOTIEROIDEISMO', 'HIPOTIREIDOISMO', 'HIPOTIROISIMO', 'HIPOTIREPODISMO', 'HIPOTIROIDIAMO', 'HIPOTIRIOIDISM', 'HIPOTIRIOI', 'HIPOTIREOIDISMIO', 'HIPOTIREOIDISIMO', 'HIPOTIROIDISNO', 'HIPOTIREOIDISMOO', 'HIPOTIREOPIDISMO', 'HIPOTIREOISDIMO', 'HIPOTIREOIDOSMO', 'HIPOTIREOIDISMPO', 'HIPOTROFIA', 'HIPOTEROEDISMO', 'HHIPOTIROIDISMO', 'HIPOTERMIA', 'IPOTIRIOIDISMO', 'HIPOTEREODIMIA', 'HIPOTERO', 'HIPOTERIODISMOE', 'HIPOTEREOID', 'HIPOTEREOEDISMO', 'HIPOTEREODISMONENFISE', 'HIPOTIEROIDISM', 'HIPOTIREIDISM', 'HIPOTIREIDIS', 'HIPOTIREID', 'HIPOTIREEIDISMO', 'HIPOTIREDOISMO', 'HIPOTIOREDISMO', 'HIPOTIOREDIASMOS', 'HIPOTEREODIS', 'HIPOTREOIDE', 'HIPOTEREIDISMO', 'HIPOTEIRODISMO', 'HIPOTEIREODISMO', 'HIPOTEIREO', 'HIPOTIROIDIDSMO', 'HIPOTIROIDOSMO', 'HIPOTIROIDISMOSMO', 'HIPOTIROIDISIMO', 'HIPOTIROIDIMOS', 'HIPOTIROIDICO', 'HIPOTIROIDESMO', 'HIPOTIROIDEO', 'HIPOTIROEDISM', 'HIPOTIROIDSIMO', 'HIPOTIROIISMO', 'HIPOTOREODISMO', 'HIPOTOREODI', 'HIPOTIROITISMO', 'HIPOTIROITIDISMO', 'HIPOTIROISDIMO', 'HIPOTIREOIDISMOI', 'HIPOTIREOIDIM', 'HIPOTIREOIDIDMO', 'HIPOTIREODIMIO', 'HIPOTIREODIDISMO', 'HIPOTIREOADISMO', 'HIPOTIREIOIDE', 'HIPOTIREOIDISMOE', 'HIPOTIRIOIDIS', 'HIPOTIRIEOIDISMO', 'HIPOTIRIEODISMO', 'HIPOTIRI', 'HIPOTIREOTISMO', 'HIPOTIREOSDISMO', 'HIPOTIREOITISMO', 'HIPOTIREOISDIS', 'HIPOTIREOIS', 'HIPOTIREOIISMO', 'HIPOTIREOIDISTO', 'HIPOTIREOIDISNO', 'HIPOITIRODISMO', 'HIPOTTIREOIDISMO', 'HIPOTTIREODISMO', 'HIPTIROIDISMO', 'HHIPOTIREOIDISMO', 'HIOPOTIROIDISMO', 'HIOPOTIRODISMO', 'IPOTIROIDISMO', 'IPOTERIODISMO', 'IPOTIREIODISMO', 'HIPOTEIREOIDE', 'HIPOTEIREOISDISMO', 'HIHPOTIREODISMO', 'HIPOITEROIDISMO', 'HIPOITREOIDISMO', 'HIPOTERAUDISMO', 'HIPOTIREOIDOISMO', 'HIPOTIREIOTISMO', 'HIPOTIREISDIMO', 'HIPOTIREISDISMO', 'HIPOTIREISMO', 'HIPOTIREOCEIDEIA', 'HIPOTIREODEA', 'HIPOTIREODIMA', 'HIPOTIREIOIDISM', 'HIPOTIREODIOSMO', 'HIPOTIREODISIMO', 'HIPOTIREODISMI', 'HIPOTIREIOIDIS', 'HIPOTEREIOD', 'HIPOTIREEODISMO', 'HIPOTIREEOIDISMO', 'HIPOTIREIDINO', 'HIPOTIREIDISMAO', 'HIPOTIREIIDISMO', 'HIPOTIREIOID', 'HIPOTIREIOCAIS', 'HIPOTIREIOD', 'HIPOTIREIODE', 'HIPOTIREIODI', 'HIPOTIREIODIDMO', 'HIPOTIREODISMOS', 'HIPOTIREODISMOTABAGIST', 'HIPOTIREODISMP', 'HIPOTIREOIDISDO', 'HIPOTIREOIDISMMO', 'HIPOTIREOIDISMOHAS', 'HIPOTIREODISMPO', 'HIPOTIREOIDISNOM', 'HIPOTIREOIDISO', 'HIPOTIREOIDISOMO', 'HIPOTIREOIDIISMO', 'HIPOTIREOIDIISM', 'HIPOTIREOIDIANO', 'HIPOTIREODISO', 'HIPOTIREODSMO', 'HIPOTIREOEDIS', 'HIPOTIREOIDEIMO', 'HIPOTIREOIDEISM', 'HIPOTIREOIDEMO', 'HIPOTIREOIDES', 'HIPOTIREOIDIAMO', 'HIPOTIREDOIDISMO', 'HIPOTEROPIDISMO', 'HIPOTERIOSMIDISMO', 'HIPOTERMICO', 'HIPOTEROID', 'HIPOTEROIDI', 'HIPOTEROIDIMOS', 'HIPOTEROISMO', 'HIPOTERIOSIMO', 'HIPOTERIORDISMO', 'HIPOTERIOEDISMO', 'HIPOTEREIOIDISMO', 'HIPOTEREODIA', 'HIPOTEREODISM', 'HIPOTEREOIDEISMO', 'HIPOTEREOIDGESTANTE', 'HIPOTEREOIDI', 'HIPOTEREOIDIS', 'HIPOTEREOIDISM', 'HIPOTEREOISDISMO', 'HIPOTIOIRIDISMO', 'HIPOTIOREODISMO', 'HIPOTIORIODISMO', 'HIPOTIORIOIDISMO', 'HIPOTIRAOIDISMO', 'HIPOTIRCODISMO', 'HIPOTIRCOIDISMO', 'HIPOTIRD', 'HIPOTIRDISMO', 'HIPOTIREAIODISMO', 'HIPOTIREDE', 'HIPOTIREDIOISM', 'HIPOTIERISMO', 'HIPOTIERORIDISMO', 'HIPOTIIREOIDI', 'HIIPOTIREOIDISMO', 'HIOPTIREOIDISMO', 'HIOPOTIREOI', 'HIPETERNSAO', 'HIPETEROIDISMO', 'HIPETERSAO', 'HIPETIORIDISMO', 'HIPETIREO', 'HIPETIREOID', 'HIPETIREOIDE', 'HIPETIREOIODISMO', 'HIPETIROEDISMO', 'HIPOATIREOIDISMO', 'HIPETIROIDI', 'HIPIOTIREOIDISMO', 'HIPOTIREOIDITE', 'HIPOTIREOIDOS', 'IHIPOTIREOID', 'HIPOTOREPODISMO', 'HIPOTOEROIDISMO', 'HIPOTORI', 'HIPOTORIDISMO', 'HIPOTORIOIDISMO', 'HIPOTOROEDISM', 'HIPOTOROIDIS', 'HIPOTOROIRISMO', 'HIPOTORPIDISMO', 'HIPOTRD', 'HIPOTIROIRISMO', 'HIPOTIROISISMO', 'HIPOTIROITID', 'HIPOTIROPI', 'HIPOTIRORIDISMO', 'HIPOTIROROI', 'HIPOTIRPIDISMO', 'HIPOTIRPODISMO', 'HIPOTRECIDISMO', 'HIPOTREIDISMO', 'HIPOTREOIDIANO', 'HIPOTRERIODISMO', 'HIPOTRIEOIDISMO', 'HIPOTRIODISMO', 'HIPOTRIREOIDISMO', 'HIPOTRIROIDISMO', 'HIPOTRODISMO', 'HIPOTROISISMO', 'HIPOTIROIEDISMO', 'HIPOTIROIDUSMO', 'HIPOTIRID', 'HIPOTIREOSDIMO', 'HIPOTIREOSIDISMO', 'HIPOTIREOSIMO', 'HIPOTIREPIDISMO', 'HIPOTIREPOIDISMO', 'HIPOTIRIDIS', 'HIPOTIROD', 'HIPOTIRIOEDISMO', 'HIPOTIRIOIDSIMO', 'HIPOTIREORISMO', 'HIPOTIREORDISMO', 'HIPOTIREOOIDISMO', 'HIPOTIREOODISMO', 'HIPOTIREOII', 'HIPOTIREOIM', 'HIPOTIREOINDISMO', 'HIPOTIREOISD', 'HIPOTIREOISDISM', 'HIPOTIREOISIDMO', 'HIPOTIREOISM', 'HIPOTIREONDISMO', 'HIPOTIRODIS', 'HIPOTIROIDISMMO', 'HIPOTIROIDIMSO', 'HIPOTIROIDISDO', 'HIPOTIROIDISLMO', 'HIPOTIROIDISMI', 'HIPOTIROIDISMIO', 'HIPOTIROIDISMNEO', 'HIPOTIRODISM', 'HIPOTIROIDISMOE', 'HIPOTIROIDISMP', 'HIPOTIROIDISMTO', 'HIPOTIROIDISOMO', 'HIPOTIROIDITE', 'HIPOTIROIDIDMO', 'HIPOTIRODISMOS', 'HIPOTIROEDIS', 'HIPOTIROEID', 'HIPOTIROEOIDISMO', 'HIPOTIROIDA', 'HIPOTIROIDEMIA', 'HIPOTIROIDES', 'HIPTIREODISMO', 'HIPTIREOIDISM'] |
| COPD | dpoc | dpoc | Medical condition | DPK | ['DPOC', 'DPCO', 'DOPC', 'DPC', 'DPCC', 'DPOOC', 'DOPOC', 'DPOUC', 'DPOCA', 'DPOCO', 'DPPOC', 'DPOCC', 'DDPOC'] |
| COPD | dpoc | enfisema | Medical condition | ENFZ | ['ENFISEMA', 'ENFIZEMA', 'ENFISEMATOSO', 'ENFESEMA', 'ENFISE', 'ENFISEM', 'ENFISEMATOSA', 'ENFISIMA', 'ENFIZEMATOSO', 'ENFIZENA', 'ENFISEMAPULMONAR', 'ENFISA', 'ENFISENA', 'ENFISEMIA', 'ENFISEMAMA', 'ENFISEMAE', 'ENFIZEMIA', 'ENFIZEMAR', 'ENFIZEM', 'ENFASENA', 'ENFESIMA', 'ENFISEMAA', 'ENFIASEMA'] |
| Alzheimer | alzheimer | alzheimer | Medical condition | AZM2 | ['ALZHEIMER', 'ALZHAIMER', 'ALZEIMER', 'ALZAIMER', 'ALZEHEIMER', 'ALZHEMER', 'ALZHEIMEIR', 'ALZHEMIER', 'ALZAHEIMER', 'ALZEIHMER', 'ALZHIMER', 'AZHEIMER', 'ALZHEMEIR', 'ALZHEIMAR', 'ALZHMER', 'ALZHEIMIER', 'ALZIMER', 'ALZHHEIMER', 'ALZEHIMER', 'ALZHIEMER', 'ALZHEYMER', 'ALZAMER', 'ALZHAMIER', 'ALZHAMER', 'ALZHAEIMER', 'ALZEIMHER', 'ALZAHIMER', 'ALZEIMEIR', 'ALZAMIER', 'ALZEHMEIR', 'ALZHEIMERR', 'ALZHEIMR', 'ALZAHEMER', 'ALZAIHMER', 'ALZHEIIMER', 'ALZZHEIMER', 'AUZHEIMER', 'ALZHMEIR', 'ALZHUMER', 'ALZIEMER', 'ALZHEEIMER', 'ALZEMER', 'ALZEMEIER', 'ALZHEMIR', 'ALZHEIMMER', 'ALZHEIMIR', 'ALZAEIMER', 'ALZAHAIMER', 'ALZAHMER', 'HALZHEIMER', 'ALZHAMEIR', 'ALZAHIEMER', 'ALZHEIUMER', 'ALZHEIMAER', 'AHZHEIMER', 'AHLZEIMER', 'ALZEILMER', 'ALZEIHEMR', 'ALZAEMER', 'ALZEMEIR', 'ALZEMHIER', 'ALZHAIMR', 'ALZHAIMEIR', 'ALZHAEMER', 'ALLZHAIMER', 'AUZHAIMER', 'AUZHHEIMER', 'ALZHMEIER', 'ALZHEMAER', 'ALZHWMER', 'ALZHHAIMER', 'ALZHUIMER', 'ALZHIMIR', 'ALZMEIR', 'ALZYEIMER', 'ALZIHEIMER', 'ALZIMEIR', 'ALZAMEIR', 'ALZAEHIMER', 'ALZAHAMER', 'ALLZHEIMER', 'ALLZEIMER', 'ALZHEIMEER', 'ALZHEMEIER', 'ALZHEMAR', 'ALZHELMAR', 'ALZEIHEMER', 'ALZEHMIER', 'ALZEHMEIER', 'ALZEHIEMER', 'ALZEHEMER', 'ALZEHAIMER', 'ALZAYMER', 'ALZHALMER', 'ALZHEILMER', 'ALZHAYMER', 'ALZEMIER', 'AHZEIMER', 'AHLZEIMEIR', 'AIZAIMER', 'AIZHEIMER', 'HALZAIMER', 'HALZIMER', 'AZHAIMER', 'AZHEIMIER', 'AUSAIMER'] |
| Alchoolism | etilista | etilista | Medical condition | ETLS | ['ETILISTA', 'ETILISMO', 'ETILIS', 'ETILIST', 'ETLISTA', 'ETILISM', 'ETLISMO', 'ETELISTA', 'ELTILISTA', 'ETILISTMO', 'EITILISTA', 'ETILISTAS', 'HETILISTA', 'ETILISTO', 'ETILISMA', 'EITLISTA', 'ETILIISTA', 'ETELISMO', 'EITLISMO', 'ETILISTAA', 'ELTILISMO', 'EETILISMO', 'ETILOSMO', 'ETILISTACRONICO', 'ETILISTATABAGISTA', 'ETILLISTA', 'ETILISTYA', 'ETILISTS', 'ETILISTRA', 'ETILISTQA', 'ETILISTAUSO', 'ETLIS', 'ETTLIS', 'ETILESTOR', 'ETILESTA', 'ETILES', 'ETIELISTA', 'ETILIESTA'] |
| Obesity | obesidade | obesidade | Medical condition | OBZD | ['OBESIDADE', 'OBESID', 'OBESIDAD', 'OBESIDADDE', 'OBESIDA', 'OBESIDADES', 'OBESIADE', 'OBESIDAE', 'OBESIDADADE', 'OBESIDDE', 'OBESIDADAE', 'OBESIDADED', 'OBESIDEDA', 'OBESIDASDE', 'OBESIADADE', 'HOBESIDADE', 'OBBESIDADE', 'OBESIDADEIMC', 'OBESIDADECANCER', 'OBESIDADEGRAU', 'OBESIDADEHIPOTIREODISMO', 'OBESIDADEID', 'OBESIDADESEM', 'OBESODADE', 'OBESIDASE', 'OBESIDSASE', 'OBESIDAADE', 'OBEISIDADE', 'OBESEIDADE', 'OBESIADDE', 'OBESUDADE', 'OBOSIDADE'] |
| Depression | depressao | depressao | Medical condition | DPRS | ['DEPRESSAO', 'DEPRESSIVO', 'DEPRESSA', 'DEPRESSIVA', 'DEPRESS', 'DEPRES', 'DEPRESSI', 'DEPRESSIV', 'DEPRESSAP', 'DEPRESSIVOS', 'DEPRESSSAO', 'DPRESSAO', 'DEPPRESSAO', 'DEPRSSAO', 'DEPRESSOA', 'DEPRESSO', 'DEEPRESSAO', 'DEPRESSAI', 'DEPRESSAOI', 'DEPORESSAO', 'DEPRASSAO', 'DEPRS', 'DEPERSSAO', 'DEPERESSAO', 'DEPREESSAO', 'DEPRESSAOARTROSE', 'DEPRESSAA', 'DEPRESSAOD', 'DEPRESSAOO', 'DEPRESSIVEX', 'DEPRESSIVEL', 'DEPRESSCAO', 'DEPRESSCA', 'DEPRESSAPO', 'DEPRESSIVOI', 'DEPRESSS', 'DEAPRESSAO', 'DIPRESSAO'] |
| Epilepsy | epilepsia | epilepsia | Medical condition | EPLP | ['EPILEPSIA', 'EPLEPSIA', 'EPILEPSI', 'EPILEP', 'EPILEPS', 'EPILEPTICO', 'EPELEPSIA', 'EPILEPTICA', 'EPILEPCIA', 'EPILIPSIA', 'EPILEPISIA', 'EPLEPISIA', 'EPLEPCIA', 'HEPILEPSIA', 'EPLIPSIA', 'EPILIEPSIA', 'EPILEPSCIA', 'EPLEPTICO', 'EPLEPS', 'EPLEPIA', 'EPLEP', 'EPELIPSIA', 'EPOLEPSIA', 'EPLEPICIA', 'EPLIPETICO', 'EPILAPSIA', 'EPILEPSIO', 'EPILEPSA', 'EPILEPISA', 'EPLLEPSIA', 'EPILEPTICAS', 'EPILEPTIOCA', 'EPILEPSIIA', 'EPILEPSO', 'EPILIPRIA', 'EPILEPSIS', 'EPILEPC', 'EPILEPISSIA', 'EPILEPPSIA', 'EPILEPSICIA', 'EPILEPSIAPARALIS', 'EPILEPSIAI', 'EPILEPRIA', 'EPILLEPSIA', 'EPLEPTICA', 'EPLEPSI', 'EPLEPISA', 'EPLIPESIA', 'EPLIPCIA', 'HEPILEP'] |
| Cardiopathy | cardiopata | cardiopata | Medical condition | KRDP | ['CARDIOPATA', 'CARDIOPATIA', 'CARDIOP', 'CARDIOPAT', 'CARDIOPA', 'CARDIOPATI', 'CARDIOPATIAS', 'CARDIOPTA', 'CARDIPATA', 'CARDIPOATA', 'CARDIPATIA', 'CARDIOPARA', 'CARDIOPADA', 'CARDIOPARIA', 'CARDIOPAIA', 'CARDIOOPATA', 'CARDIOAPATIA', 'CARDIAOPATIA', 'CARDIPOPATA', 'CARDIOPATICO', 'CARDIOPATO', 'CARDIOPTIA', 'CRADIOPATIA', 'CRDIOPATA', 'CORDIOPATA', 'CARDIOPATAC', 'CARDIOPATAS', 'CARDIOPALTA', 'CARDIOPAATIA', 'CARDIOPAA', 'CARDIAPATIA', 'CARDEOPOTA', 'CARDIIOPATA', 'CARDIOPLA', 'CARDIOPENIA', 'CARDIOPIA', 'CARDIOPATICAS', 'CARDIOPATIAIAM', 'CARDIOPATIACONGENITA', 'CARDIOPATITE', 'CARDIP', 'CARDIPOATIA', 'CARIDOPATIA'] |
| Cardiopathy | cardiopata | ic | Medical condition | IK | ['ICC', 'IC'] |
| Parkinson | parkinson | parkinson | Medical condition | PRKN | ['PARKINSON', 'PARKINS', 'PARKINSONISMO', 'PARKINSO', 'PARKINSSON', 'PARKNSON', 'PARKINSOM', 'PARKENSON', 'PARCKINSON', 'PARKINGSON', 'PARKINSONIANA', 'PARKINSIN', 'PERKINSON', 'PARQUINSON', 'PARKNISO', 'PARKINSONS', 'PARKIINSON', 'PRKINSON', 'PORKINSO', 'PARKNSONIANA', 'PARKNISON', 'PARKEINSON', 'PARKENNSON', 'PARKENSOU', 'PARKINSONIS', 'PARKINSONM', 'PARKINSOR', 'PARKINSS', 'PARKINSSONISMO', 'PARKIONSON', 'PARKKINSON', 'PARKINSONIANO', 'PARKINSONIAMA', 'PARKHINSON', 'PARKINAON', 'PARKINGON', 'PARKINGSO', 'PARKINGSSON', 'PARKINISMO', 'PARKINISON', 'PARKINKSON', 'PARKINNSON', 'PARKINOON'] |
| Schizophrenia | esquizofrenia | esquizofrenia | Medical condition | ESKZ | ['ESQUIZOFRENIA', 'ESQUISOFRENIA', 'ESQUIZOFRENICO', 'ESQUIZO', 'ESQUIZOF', 'ESQUIZOFRENI', 'ESQUIZOFREN', 'ESQUIZOFRENICA', 'ESQUIZOFR', 'ESQUISOFRENICO', 'ESQUIZOFENIA', 'ESQUIZOENCEFALIA', 'ESQUEZOFRENIA', 'ESQUIZOFRE', 'ESQUIZENCEFALIA', 'ESQUIZOAFETIVO', 'ESQUISOFRENIC', 'ESQUIZOFRINIA', 'ESQUIZOFREMIA', 'ESQUIZOFRENIO', 'ESQIOZOFRENIA', 'ESQUIZIOFRENIA', 'ESQUIZIFRENIA', 'ESQUISOFREN', 'ESQUIZOFRI', 'ESQUIZONEFRENIA', 'ESQUIZOOFRENIA', 'ESQUZOFRENIA', 'ESQUZOFRENCIA', 'ESQUEZOFRENA', 'ESQUISOFENIA', 'ESQIZOFRENIA', 'ESQUIZAOFRENIA', 'ESQUIZAFRENIA', 'ESQUISOFREMIA', 'ESQUISOFR', 'ESQUISOFRENICA', 'ESQUIZFRENIA', 'ESQUIZOFRENIS', 'ESQUIZOENCEFA', 'ESQUIZOFREINA', 'ESQUIZOFRENEIA', 'ESQUIZPFRENIA', 'EASQUIZOFRENIA', 'ESQUIZOFFRENIA', 'ESQUIZOFERNIA', 'ESQUIZEFRINIA', 'ESQUIZAFONIA', 'ESQUIZOFRENCO', 'ESQUIZOFRENCIA', 'ESQUIZOFRENA', 'ESQUIZOFRANIA', 'ESQUISOFROMIA', 'ESQUISOFRENIO', 'ESQUISOFRENIIA', 'ESQUZOFRINIA', 'ESQUOZOF', 'ESQUIZOFRONIA', 'ESQUIZOFRIA', 'ESQUIZPFRENICO', 'ESQUIZOFRENUA', 'ESQUIZOFRENO', 'ESQUIZOFRRENIA', 'ESQUIZORENIA', 'ESQUIZOPOFRENIA', 'ESQUIZOPFRENIA', 'ESQUIZONFRENIA', 'ESQUISOF', 'ESQUISIOFRENIA', 'ESQUISIFRENIA', 'ESQUISOFRENI', 'ESQUISOFRE', 'ESQUISOFFRENIA', 'ESQISOFRENIA', 'ESQUEIZOFRENIA', 'ESQUEZOFRENICO', 'ESQUEZOFRENICA'] |
| Limphoma | linfoma | linfoma | Medical condition | LNFM | ['LINFOMA', 'LONFOMA', 'LINFOMAS', 'LINFOM', 'LINFOME', 'LINFOMO', 'LIINFOMA', 'LINFOMEGALIA', 'LINFOMAN', 'LINFOMADO', 'LILNFOMA', 'LINFIMA', 'LINFAM', 'LINFAN', 'LNFOMA', 'LINFOMAHODGKINAOS', 'LINFOMAR', 'LINFOMODOS', 'LINFON', 'LINFOMAGASTRICO'] |
| Rheumatoid arthritis | artrite reumatoide | artrite reumatoide | Medical condition | ARTR 2MTD | ['ARTRITE REUMATOIDE', 'ARTRITE REMATOIDE', 'ARTRITE REUMATOID', 'ARTRITE REUMTOIDE', 'ARTRITE REMAUTOIDE', 'ARTRIDE REUMATOIDE', 'ARTRITE RELMATOIDE', 'ARTRITR REUMATOIDE', 'ARTRITE REUMATODE', 'ARTRIT REUMATOIDE', 'ARTRIRE REUMATOIDE', 'ARTTRITE REUMATOIDE', 'ARTROTE REMAUTOIDE', 'ARTRITE REUMAITOIDE', 'ARTRITE RUMATOIDE', 'ARTRITE REUMATOLDE', 'ARTRITE REUMATIDE', 'ARTRITE REUMATOIDA', 'ARTRITITE REUMATOIDE', 'ARTRITRE REUMATOID', 'ARTRITRE REUMATOIDE', 'ARTRITE RUIMATOIDE', 'ARTRITER REUMATOIDE', 'ARTRITI REUMATOIDE', 'ARTRITE REMALTOIDE', 'ARTRITE REMAITODE', 'ARTRITE RELMOTODE', 'ARTRITE REAUMATOIDE', 'ARTRITE REOMATOIDE', 'ARTRITE RAMAUTOIDE', 'ARTRITE RAMATOIDE', 'ARTRITE REUMATOD', 'ARTRITE REUMATOOIDE', 'ARTRITE REUMATOIDO', 'ARTRITE REUMATOIDECA', 'ARTRITE REUMATODOITE', 'ARTRITE REUMATIODE', 'ARTRITE REUMALTOIDE', 'ARTIRITE REUMATOIDE', 'ARTRITA REUMATOIDE', 'ARTRISTE REUMATOIDE', 'ARTROSE REUMATOIDE', 'ARTRTITE REUMATOIDE', 'ARTROTE REUMATOIDE'] |
| Breast cancer | cancer de mama | ca mama | Medical condition | K MM | ['CA MAMA', 'CC MAMA', 'CO MAMA', 'C MIOMA', 'CA MAN', 'CA MOMA', 'CA MAMAE'] |
| Breast cancer | cancer de mama | ca de mama | Medical condition | K D MM | ['CA DE MAMA', 'CA DA MAMA', 'CA DE MAM', 'CA D MAMA', 'CL DE MAMA'] |
| Breast cancer | cancer de mama | cancer de mama | Medical condition | KNS2 D MM | ['CANCER DE MAMA', 'CANCER DE MAM', 'CAANCER DE MAMA', 'CNCER DE MAMA', 'CANCER DA MAMA', 'CANCER D MAMA'] |
| Breast cancer | cancer de mama | neoplasia de mama | Medical condition | NPLZ D MM | ['NEOPLASIA DE MAMA', 'NEOPLASIA DA MAMA', 'NEOPLAISA DE MAMA', 'NEOPLASI DE MAMA', 'NEOPLASIA DE MAM', 'NOPLASIA DE MAMA', 'NEPLASIA DE MAMA', 'NEOPLASIAS DE MAMA'] |
| Asthma | asma | asma | Medical condition | ASM | ['ASMA'] |
| Asthma | asma | bronquite | Medical condition | BRNK | ['BRONQUITE', 'BRONQUIT', 'BRINQUITE', 'BRANQUITE', 'BRONQUITES', 'BRONQUINTE', 'BRONQUITI', 'BRONQUIETE', 'BRONQUT', 'BRIONQUITE', 'BRINQUIT', 'BORNQUITE'] |
| Rhinitis | rinite | rinite | Medical condition | RNT | ['RINITE', 'RENITE', 'RINITI', 'RENITI', 'RENIT', 'RINIT', 'RNITE'] |
| Chronic kidney disease | doenca renal cronica | doenca renal cronica | Medical condition | DNK 2N KRNK | ['DOENCA RENAL CRONICA', 'DOENCA RENAL CRONIC'] |
| Hyperthyroidism | hipertireoidismo | hipertireoidismo | Medical condition | IPRT | ['HIPERTIREOIDISMO', 'HIPERTIROIDISMO', 'HIPERTIREODISMO', 'HIPERTIREO', 'HIPERTIREIODISMO', 'HIPERTIREOIDISM', 'HIPERTIRIODISMO', 'HIPERTIREOIDE', 'HIPERTEREOIDISMO', 'HIPERTIR', 'HIPERTIREOIDIS', 'HIPERTIROEDISMO', 'HIPERTIREOIDI', 'HIPERTIREOID', 'HIPERTIREIDISMO', 'HIPERTIREIOIDISMO', 'HIPERTIREOISIMO', 'HIPERTIRIOIDISMO', 'HIPERTIROIDIS', 'HIPERTIREOI', 'HIPERTIREOIDISMOS', 'HIPERTIRODISMO', 'HIPERTIREOISDISMO', 'HIPERTEROIDISMO', 'HIPERTIOIDISMO', 'HIPERTEOIDISMO', 'HIPERTIROIDI', 'HIPERTIEOIDISMO', 'HIPERTIROID', 'HIPERTIREOIDEA', 'HIPERTIREODE', 'HIPERTIRE', 'HIPERTREOIDISMO', 'HIPERETIREOIODISMO', 'HIPERTIODISMO', 'HIPERTIORIDISMO', 'HIPIERTIREOIDISMO', 'HIPIRITIOIDISMO', 'HIPERTYIREOIDISMO', 'HIPERTIORISMO', 'HIPERTIREOIDSIMO', 'HIPERTIREOISMO', 'HIPERTIREORDISMO', 'HIPERTIREOTISMO', 'HIPERTIREPOIDISMO', 'HIPERTIRO', 'HIPERTIROIDISM', 'HIPERTIREOIDEISMO', 'HIPERTIREOIDEIA', 'HIPERTIRADISMO', 'HIPERTIRD', 'HIPERTIREIDISM', 'HIPERTIREIODOSMO', 'HIPERTIREOD', 'HIPERTIREODI', 'HIPERTIREODIS', 'HIPERTIREODSIMO', 'HIPERTUROIDISMA', 'HIPERTOROIDISMO', 'HIPERTR', 'HIPERTRAODISMO', 'HIPERTREODISMO', 'IHPERTIREOIDISMO'] |
| Pneumonia | pneumonia | pneumonia | Medical condition | PNMN | ['PNEUMONIA', 'PNEUMONI', 'PNEUMONIAS', 'PNEMONIA', 'PENUMONIA', 'PENEUMONIA', 'PNEUMINIA', 'PNEOMUNIA', 'PNEUMUNIA', 'PNEUMANIA', 'PNEMONIAS', 'PNEOMONIA', 'PNEUMONIAL', 'PNUEMONIA', 'PNEUMMONIA', 'PNAMUNIA', 'PNEMUNIA', 'PENAMUNIA', 'PENUEMONIA'] |
| Headache | cefaleia | cefaleia | Symptom | SFL | ['CEFALEIA', 'CEFALIA', 'CEFALEI', 'CEFALE', 'CEFALEA', 'CEFELEIA', 'CEFLEIA', 'CEFAELIA', 'CEFALAEIA', 'SEFALEIA', 'CEFALIEA', 'CEFLAEIA', 'CEFFALEIA', 'CEFALLEIA', 'CEAFALEIA', 'CEEFALEIA', 'CEFALEIAE', 'CEFELIA', 'CEFALEAI', 'CEFALAIA', 'CEFALEIIA', 'CEFALEEIA', 'CEFEALEIA', 'CEFAELEIA', 'CEAFLEIA', 'CEFALEIO', 'CELFALEIA', 'CEFALEAIA', 'CEFALEOA', 'CEFALEIAA', 'CEFAALEIA', 'CEFALI', 'CCEFALEIA', 'CEFALEIAL', 'CIFALEIA', 'CEWFALEIA', 'CEFELAIA', 'CEFALEIAI', 'CEFALEO', 'CEFALEIUA', 'CEFAELE', 'CEFAELAI', 'SEFALIA', 'CEFALEIEA', 'CEFALEIE', 'CEFALIO', 'CEFALIEIA', 'CEFALUA', 'CEFALOEIA', 'CEFALIE', 'CEFALEUA', 'CEFALEUIA', 'CEFALEE', 'CEFALAIE', 'CEFALAI', 'CEFAELA', 'CEFALA', 'CIFALIA', 'CIFELIA', 'CEFOLEIA', 'CEFLEIAA', 'CEFLE', 'CEFLAIE', 'CEFELAEIA', 'CEFFALIA', 'CEAFELIA'] |
| Headache | cefaleia | dor de cabeca | Symptom | D2 D KBK | ['DOR DE CABECA', 'DOR DE CABEC', 'DOR D CABECA', 'DOR DA CABECA', 'DOR DE CBECA', 'DOR DE CAEBCA', 'DOR DE CABACA', 'DOR DE CABEACA', 'DIR DE CABECA', 'DOIR DE CABECA', 'DOR DE CABECO', 'DOR DE CABCA', 'DDOR DE CABECA'] |
| Headache | cefaleia | dor na cabeca | Symptom | D2 N KBK | ['DOR NA CABECA', 'DOR NO CABECA'] |
| Myalgia | mialgia | mialgia | Symptom | MJ | ['MIALGIA', 'MIALGI', 'MILGIA', 'MALGIA', 'MIAGIA', 'MAILGIA', 'MIAUGIA', 'MEALGIA', 'MIOLGIA', 'MIIALGIA', 'MAIALGIA', 'MIALGIOA', 'MIALGIIA', 'MIOALGIA', 'MIAGIAL', 'MIALGIAE', 'MIALGIUA', 'MIALLGIA', 'MIALGIO', 'MIELGIA', 'MUALGIA', 'MIOGIA', 'MIAGI', 'MELGIA', 'MIALGIAL', 'MIALGIAA', 'MMIALGIA', 'MIALGEIA', 'MIAALGIA', 'MIOGIL', 'MIUALGIA', 'MILGI', 'MIALGHIA', 'MIALGGIA', 'MIALJIA', 'MIALGIE', 'MAIGIA', 'MIALGHI', 'MGE', 'MALGEO', 'MALGIIA', 'MJE', 'MOAILGIA', 'MIALGIL', 'MIALGIAI', 'MIEALGIA', 'MUIALGIA', 'MUALGI'] |
| Myalgia | mialgia | dor no corpo | Symptom | D2 N KRP | ['DOR NO CORPO', 'DOR NO CORP', 'DOR NO CARPO', 'DR NO CORPO', 'DOR NO CORPA', 'DOR NA CORPO', 'DOR NA CARPA', 'DOR NO CORPP', 'DOR NO CORPOO', 'DOR NO CORPOE'] |
| Runny nose | coriza | coriza | Symptom | KRZ | ['CORIZA', 'CORZA', 'CORIZE', 'CROIZA', 'CARIZA', 'CCORIZA', 'CRIZA', 'CORIZZA', 'COROZA', 'CORIZAA', 'CORIIZA', 'CORIZO', 'COORIZA', 'CORIZAE', 'COREZA', 'CORUIZA', 'CORZIA', 'COROIZA', 'COIRIZA', 'COIRZA', 'COREIZA', 'CORIAZA', 'CORIOZA', 'CORIZAI', 'CROZA'] |
| Asthenia | astenia | astenia | Symptom | ASTN | ['ASTENIA', 'ASTENI', 'ASTENIAS', 'ASTINIA', 'ASTNIA', 'ASTENIO', 'ASTENICO', 'ASTONIA', 'ASTENIS', 'ASTENA', 'ASTENICA', 'AASTENIA', 'ASTANIA', 'ASSTENIA', 'ASTNEIA', 'ASTENIIA', 'ASTENIAE', 'ASTEINIA', 'ASTENAI', 'ASTENIAM', 'ASTENEIA', 'ASTENAIA', 'ASTENIAMIALGIA', 'ASTENIAARTOPNEIA', 'ASTENIOA', 'ASTENIE', 'ASTENIASE', 'ASTENEA', 'ASTENCA', 'ASTENAL', 'ASTEWNIA', 'ASTINEIA', 'HASTENIA', 'AUSTENIA', 'ASSITINTOMATICA', 'ASTENCIA', 'ASTENIAAGEUSIA', 'ASTENIAAFASIA', 'ASTENIAA', 'ASTEINA', 'ASTENBIA', 'ASTENIACALAFRIOSPOLIARTRAGIA', 'ASTTENIA', 'ASTINA', 'ASTINEO', 'ASTNENIA', 'ASWTENIA', 'ASTENIACORIZA', 'ASTENINAS', 'ASTENINA', 'ASTENIC', 'ASTENIATONTURA', 'ASTENNIA', 'ASTENMIA', 'ASTENISA', 'ASTENINIA', 'ASTENIAL', 'ASTENIAFRAQUEZA', 'ASTENIADOR', 'ASTENIADIS', 'ASTENIAMIALGIAAFASIARNC', 'ASTENIAQ', 'ASTENIANO', 'ASTENIAMIALGIATONTURA', 'ASTENOA', 'ASTENSAO', 'ASTENOSE', 'ASTENOIA', 'ASTENTIA'] |
| Asthenia | astenia | fraqueza | Symptom | FRKZ | ['FRAQUEZA', 'FRAQUESA', 'FRAQUZA', 'FRQUEZA', 'FARQUEZA', 'FRAQEUZA', 'FRAQUEZAS', 'FREQUEZA', 'FRAQEZA', 'FRAAQUEZA', 'FRAQQUEZA', 'FRAQIEZA', 'FRAUQEZA', 'FRAQUAZA', 'FRAQUEZZA', 'FRAQUEZAQ', 'FRAQUSA', 'FRAQUEZQ', 'FRAQUEZAM', 'FREAQUEZA', 'FRAUQUEZA', 'FRQUZA', 'FRQZA', 'FRAQUESAS', 'FRAQUEZAINAPETENCIA', 'FRAQZA', 'FRAQUEZAN', 'FRAQUEZAPROSTACAO', 'FRAQUIZA', 'FRAQUWZA', 'FARQEUZA', 'FARAQUEZA', 'FFRAQUEZA', 'FRAQUESACEFALEIA', 'FRAQUEZAASTENIA', 'FRAQUEZACANSACO', 'FRAQUEZAENJOO', 'FRAQUEZAEZA', 'FRAQUEZAMIALGIA', 'FRAQUEZAMMII', 'FRAQUEZAMUSCULAR', 'FRAQUEZANAUSEAS', 'FRAQUEAZA', 'FRAQEUSA', 'FRAQUEZE', 'FRAQUEZEA', 'FRAQUUEZA', 'FRAQUYEZA', 'HFRAQUEZA'] |
| Asthenia | astenia | prostracao | Symptom | PRST | ['PROSTRACAO', 'PROSTACAO', 'PROSTRADO', 'PROSTRADA', 'PROSTRACA', 'PROST', 'PROSTRA', 'PROSTADO', 'PROSTRAC', 'PROSTR', 'PROSTADA', 'PROSTAC', 'PROSTACA', 'PROSTCAO', 'PRSTRACAO', 'PRSOTACAO', 'PORSTRACAO', 'PORSTACAO', 'PROSTATACAO', 'PROSTACO', 'PROSTALGIA', 'PROSTATICA', 'PRSOTRACAO', 'PROSTIACAO', 'PROSTRACAOSONOLENCIAMI', 'PARESTES', 'PPROSTRACAO', 'PROSTECAO', 'PROSTEACAO', 'PROSTACOES', 'PROSTRACOES', 'PROSTRATICA', 'PROSTRACO', 'PROSTRACAP', 'PROSTRACAOI', 'PROSTACOA', 'PROSTACAP', 'PROSTRTACAO', 'PRSTACAO', 'PROESTACAO', 'PROLSTRACAO', 'PROOSTRACAO', 'PROSTRACOO', 'PROSTRACOA', 'PROSTRACAOSINT', 'PROSTRACAOQ', 'PROSTRACAONAUSEA', 'PROSTRACAODELIRIUM', 'PROSTRACAOA', 'PROSTRACAI', 'PROSTTRACAO', 'PROSTRSCAO', 'PROSTRATICO', 'PROSTRATADO', 'PROSTRAO', 'PROSTRATACAO', 'PROSTRATA', 'PROSTRASTACAO', 'PROSTRASCAO', 'PROSTINACAO', 'PROSTAD', 'PROSTACIO', 'PROSTACCAO', 'PROSTACAOINAPETENCIA', 'PROSTACAODOR', 'PROSTACACAO', 'PROSTAAO', 'PROSTAACAO', 'PROSTATO', 'PROSTASAO', 'PROSTARCAO', 'PROSTARADA', 'PROSTARACAO', 'PREOSTRACAO', 'PRESSAOTORAX', 'PRESTRACAO', 'PRISTACAO', 'POROSTACAO'] |
| Nausea | nauseas | nauseas | Symptom | NZS | ['NAUSEAS', 'NASAIS', 'NAUSES', 'NAUZEAS', 'NEUSEAS', 'NASEAS', 'NAUSAS', 'NAUSIAS', 'NUSEAS', 'NAUSEUAS', 'NASEUAS', 'NAAUSEAS', 'NAUSEAAS', 'NAUESEAS', 'NAUSEIAS', 'NUASEAS', 'NASIAS', 'NUSES', 'NAISEAS', 'NEUZEAS', 'NNAUSEAS', 'NAUSEAUS', 'NAUSEOS', 'NAUSEOUS', 'NEUSES', 'NAIUSEAS', 'NASAS', 'NASASL', 'NAUSAEAS', 'NAUSAES', 'NAUESAS', 'NASUEAS'] |
| Nausea | nauseas | nausea | Symptom | NZ | ['NASAL', 'NAUSEA', 'NASA', 'NAUSE', 'NAZAL', 'NAUSIA', 'NAUZEA', 'NEUSEA', 'NAUSA', 'NUSEA', 'NAUSAE', 'NASEA', 'NASEUA', 'NASAIA', 'NAUZIA', 'NAUSAEA', 'NASAU', 'NALZIA', 'NOUSEA', 'NEAUSEA', 'NAUSEU', 'NAUZA', 'NAZA', 'NEUASEA', 'NUASEA', 'NUAUSEA', 'NASAI', 'NAAUSEA', 'NAEUSEA', 'NAUESEA', 'NAUSAL', 'NAUESE', 'NASO', 'NASUEA'] |
| Fatigue | fadiga | fadiga | Symptom | FDG | ['FADIGA', 'FADIG', 'FDIGA', 'FDIAGA', 'FADOGA', 'FADUGA', 'FADIAGA', 'FADGA'] |
| Loss of appetite | inapetencia | inapetencia | Symptom | INPT | ['INAPETENCIA', 'INAPTENCIA', 'INAPET', 'INAPETENCI', 'INAPETENC', 'INAPETEN', 'INAPETE', 'INAPETENTE', 'INOPETENCIA', 'INAPATENCIA', 'INPETENCIA', 'IANPETENCIA', 'INAPETECIA', 'INAPOTENCIA', 'INAPETENICA', 'INAPITENCIA', 'INAPETENIA', 'INAPETENSIA', 'INAPETNCIA', 'INAPETEIA', 'INEPETENCIA', 'HINAPETENCIA', 'INAPT', 'INAPETANCIA', 'INAPETENCIAS', 'INAPETENCA', 'INAPTENSIA', 'INAPETEMCIA', 'INAPTE', 'IANAPETENCIA', 'INOPOTENCIA', 'INPAETENCIA', 'INAPETENCIAA', 'INAPTENC', 'INAPETINENCIA', 'INAPETENCAI', 'INAPETENICIA', 'INPATENCIA', 'INAPETERNCIA', 'INAPETENCIE', 'INOPETENCI', 'INAPTETENCIA', 'INAPPETENCIA', 'INAPETEENCIA', 'INAPETEC', 'INAPATEN', 'INAPAETENCIA', 'INAPETENCIACEFALEIA', 'INAPETENCIAE', 'INAPETENCCIA', 'INAPETECNIA', 'INAPETRENCIA', 'INAPETENNCIA', 'INAPETENDIA', 'INAPETNECIA', 'INAPATENC', 'IINAPETENCIA', 'INAPITENCI', 'INAPOTENCI', 'INAPTEN', 'INAPTENCI', 'INAPTENTE', 'INAPWTENCIA', 'INAPETENCIO', 'INAPETENEIA', 'INAPETENTENCIA', 'INAPETEWNCIA', 'INAPETEXIA', 'INAPETITE', 'IONAPETENCIA', 'INAPEETENCIA', 'INAPETENCIAM', 'INAPETENCIAN', 'HINAPETENCI', 'HINAPITENCIA', 'HINOPETENCIA', 'INAPETENCIDA', 'INAPETENCIIA', 'INAPETENCIOA', 'INAPETENCIS', 'INAPETENCO', 'INAPETENCOA', 'INAPETENSI', 'INAPETENCIAPETEQUIAS', 'INAPETENTCIA', 'INAPETERICIA', 'INAPETETNCIA', 'INAPETEUTA', 'INAPETIA', 'INAPETICAO', 'INAPETENCIAPROSTRACAO', 'INAPETENCIAODINOFAGIA', 'INAPETENCAO', 'INAPETEACIA', 'INAPETECENCIA', 'INAPETECI', 'INAPETEENCI', 'INAPETENCE', 'INAPETENCIANAUSEAS', 'INAPETENCFIA', 'INAPETENCIAASTENIA', 'INAPETENCIADOR', 'INAPETENCIAL', 'INAPETENCIAMAL', 'INAPETENCIAMIALGIA', 'INAPETINCIA', 'INAPETINECIA', 'INAPETINIA', 'INAPETN', 'INAPUTENCIA', 'INAPETNIA', 'INAPETPENCIA', 'INAPETUNA', 'INAPITE', 'INAPITERICIA', 'INAPOTENIA', 'INAPTECENCIA', 'INAPTECNIA', 'INAPTENCIAA', 'INAPTICAO', 'INAPATECIA', 'INAPATANCIA', 'INAOPETENCIA', 'INAPAT', 'INPET', 'INOPETENTE', 'INOPOT', 'INOPTENCIO', 'INPAETENCI', 'INPOTENCIA', 'INOPETENA', 'INPTCIA', 'INOPETENCIANFRAQUEZA', 'INOPETANCIA', 'INIPTENCIA', 'INEPTINEIA', 'INEPTENCIA', 'IANEPTENCIA', 'IANPATENCIA', 'IANPETECIA', 'IANPTENCIA'] |
| Loss of appetite | inapetencia | perda do apetite | Symptom | PRD D APTT | ['PERDA DE APETITE', 'PERDA DO APETITE', 'PERDE DE APETITE', 'PERDA DE APETIT', 'PERDA DO APETIT', 'PERDA DE APETITI', 'PERDA DA APETITE', 'PERDA DE APATITE', 'PERDA DE APTITE', 'PERDA DE APITITE', 'PERDA D APETITE', 'PERDA DA APETITI', 'PERDA DO APTITE', 'PERDA DE APATITI', 'PERDA DE APETITT', 'PERDA DE APETITW', 'PERDA DE APETITITE', 'PERDA DE APETITEE', 'PERDA DE APETITA', 'PERD DE APETITE', 'PERD DO APETITE'] |
| Loss of appetite | inapetencia | falta de apetite | Symptom | FT D APTT | ['FALTA DE APETITE', 'FALTA DE APETIT', 'FALTA DE APATITE', 'FALTA DE APITITE', 'FALTA DE HAPETITE', 'FALTA DO APETITE', 'FATA DE APETITE', 'FALTA DE APAETITE', 'FALTA D APETITE', 'FALTA DE APETITTE', 'FALTA DE APETITR', 'FALTA DE APETITI', 'FALTA DE APETITA', 'FALT DE APETIT', 'FALTA DE APTITE', 'FALTA DE APETITIE', 'FALTE DE APETITE'] |
| Loss of appetite | inapetencia | hiporexia | Symptom | IPRX | ['HIPOREXIA', 'HIPOREX', 'HIPOREXI', 'HIPOREXEMIA', 'HIPEREXIA', 'HIPOREXIMIA', 'HIPREXIA', 'HIPORAXIA', 'HIPOREXA', 'HIPORIXIA', 'HIPIREXIA', 'HIPORXIA', 'HIPAREXIA', 'HIPEROXIA', 'HIPOREXIO', 'HIPOREXIMA', 'HIPOREXAI', 'HIOPOREXIA', 'HIPPOREXIA', 'HIOPREXIA', 'HIIPOREXIA', 'IPOREXIA', 'HIPIOREXIA', 'HIPIROXIA', 'HIPORX', 'HIPOROXIA', 'HIPOREXCIA', 'HIPOREXEIA', 'HIPOREXENIA', 'HIPOREXIAL', 'HIPOREXICA', 'HIPOREXIS', 'HIOPEREXIA', 'HIPAREXIMIA', 'HIPIOREX', 'HIPEREXEMIA', 'HIPOREXIAPERDA', 'HIPOREXIASUDORESE', 'HIPOREXIAX', 'HIPOREXINA', 'HIPOREXOA', 'HIPORIXEMIA', 'HIPOREXIANAUSEA', 'HIPOREXIAESU', 'HIPOREXIADOR', 'HIPOREEXIA', 'HIPOREXEM', 'HIPOREXEMI', 'HIPOOREXIA', 'HYPOREXIA', 'HIPROXEIA', 'HIPROXEMIA', 'HIPROXIA', 'HIPPREXIA', 'HIPPOREXI', 'HIPPOREXA'] |
| Neurological alterations | alteracoes neurologicas | desorientacao | Symptom | DZRN | ['DESORIENTACAO', 'DESORIENTADO', 'DESORIENTADA', 'DESORIENTAC', 'DESORIENT', 'DESORIENTACA', 'DESORIENTA', 'DESORIENTCAO', 'DESORIENTAD', 'DESORINTACAO', 'DOSORIENTACAO', 'DESORIENTACAP', 'DESOREINTADA', 'DESORIENTACOES', 'DESORIENTECAO', 'DESORINETACAO', 'DESORIWNRCO', 'DESOREINTACA', 'DESOREINTACAO', 'DESOREINTADO', 'DESORIANTACAO', 'DESORIENRTACAO', 'DESORIENTACCAO', 'DESORIENTALCAO', 'DESORIENTANDO', 'DISORIENTACAO', 'DESOREIENTACAO', 'DESERENTACAO', 'DESIORIENTACAO'] |
| Neurological alterations | alteracoes neurologicas | confusao mental | Symptom | KNFZ MNT | ['CONFUSAO MENTAL', 'CONFUSAO MENT', 'CONFUSAO MENTA', 'CONFUSO MENTAL', 'CONFISAO MENTAL', 'CUNFUSAO MENTAL', 'CONFUSA MENTAL', 'CONFUZAO MENTAL', 'CONFUSAO MENTAO', 'CONFUSAO MANTAL', 'CONFUSAO MENATAL', 'CONFUSAO MENTL', 'CONFUSAO MEWNTAL', 'CONFUSAO MMENTAL', 'CONFUSAO MNETAL', 'CONFFUSAO MENTAL', 'CONFUSAL MENTAL', 'CONFUSAIO MENTAL', 'CONFUSAAO MENTAL', 'COONFUSAO MENTAL', 'CONUFUSAO MENTAL', 'CNFUSAO MENTAL', 'CCONFUSAO MENTAL'] |
| Neurological alterations | alteracoes neurologicas | confusao | Symptom | KNFZ | ['CONFUSAO', 'CONFUSO', 'CONFUSA', 'CONFUSIONAL', 'CUNFUSAO', 'CONFISAO', 'CONFUZAO', 'CONUFUSAO', 'CONFUZIONAL', 'CONNFUSAO', 'COONFUSAO', 'CONFUZA', 'CONFUSAL', 'CONFUSAAO', 'CONFUSAIO', 'CONFUSAOG', 'CONFUSIO', 'CONFUSOA', 'CONFFUSAO', 'CNFUSAO', 'CANFUSAO', 'CUNFUSA', 'CCONFUSAO'] |
| Neurological alterations | alteracoes neurologicas | amnesia | Symptom | AMNZ | ['AMNESIA', 'AMINESIA', 'AMINASEIA', 'AMNESI'] |
| Neurological alterations | alteracoes neurologicas | perda de memoria | Symptom | PRD D MMR | ['PERDA DE MEMORIA', 'PERDA DA MEMORIA'] |
| Neurological alterations | alteracoes neurologicas | esquecimento | Symptom | ESKS | ['ESQUECIMENTO', 'ESQUECIMEN', 'ESQUECIMENTOS'] |
| Neurological alterations | alteracoes neurologicas | confusao pos ictal | Symptom | KNFZ PS IKT | ['CONFUSAO POS ICTAL'] |
| Neurological alterations | alteracoes neurologicas | deficit de memoria | Symptom | DFST D MMR | ['DEFICIT DE MEMORIA'] |
| Loss of smell | anosmia | anosmia | Symptom | ANSM | ['ANOSMIA', 'ANOSMI', 'ANOSM', 'ANESMIA', 'ANASMIA', 'ANEUSMIA', 'ANSOMIA', 'ANOESMIA', 'ANOISMO', 'ANOSMA', 'ANOSMIO', 'ANOSMIG', 'ANOSMIAS', 'ANOSMIMA', 'ANOSMIS', 'ANOSMIAM', 'ANIOSMIA', 'ANISMIA', 'ANOOSMIA', 'ANOSMEIA', 'ANOSMIOA', 'ANOSMOA', 'ANOSMINIA', 'ANOSMIACORIZA', 'ANOSMIACEFALEIAMIALGIACORIZA', 'ANOSMIAAGEUSIA', 'ANOSMICE', 'ANOSMIAI', 'ANSIMA'] |
| Loss of smell | anosmia | perda de olfato | Symptom | PRD D OFT | ['PERDA DE OLFATO', 'PERDA DO OLFATO', 'PERDA DE OFATO', 'PERDA DO OFATO', 'PERDA DE OFALTO', 'PERDE DE OLFATO', 'PERDA DE OLFAT', 'PERDA D OLFATO', 'PERDE DO OLFATO', 'PERDA DO OLFAT', 'PERDA DO OLFALTO', 'PERDA DE OOLFATO', 'PERDA DE OFFATO', 'PEERDA DE OLFATO'] |
| Loss of taste | ageusia | ageusia | Symptom | AJZ | ['AGEUSIA', 'AUGESIA', 'ALGESIA', 'AGESIA', 'AGEUSI', 'AGEUSA', 'AGEOSIA', 'AUGEUSIA', 'AGEUZIA', 'ALGEZIA', 'AGIOSIA', 'AGEUSEIA', 'AGEUZI', 'AJOSIA', 'AGEUSUA', 'ALGEUSIA', 'AGEISA', 'AGEUSEA', 'AGEUSIO', 'AGEUISI', 'AGEUSAI', 'AUGISIA'] |
| Loss of taste | ageusia | perda do paladar | Symptom | PRD D PLD2 | ['PERDA DE PALADAR', 'PERDA DO PALADAR', 'PERDA DE PLADAR', 'PERDA DO PELADAR', 'PERDA DO PALADAAR', 'PERDA DA PALADAR', 'PERDA D PALADAR', 'PERDA DE PALADARR', 'PERDA DE PELADAR', 'PERDO DO PALADAR', 'PERDDA DE PALADAR'] |
| Loss of taste | ageusia | falta de paladar | Symptom | FT D PLD2 | ['FALTA DE PALADAR', 'FALTA DO PALADAR'] |
| Cough | tosse | tosse | Symptom | TS | ['TOSSE', 'TSSE'] |
| Fatigue | cansaco | fadiga | Symptom | KNSK | ['CANSACO', 'CANSAC', 'CONSACO', 'CANSCO', 'CNSACO', 'CANSSACO', 'CANSACAO', 'CANSACOS', 'CANSACCO', 'CAANSACO', 'CONSAC', 'CONCIEC', 'CANSOCO', 'CANSCOA', 'CANSACOO'] |


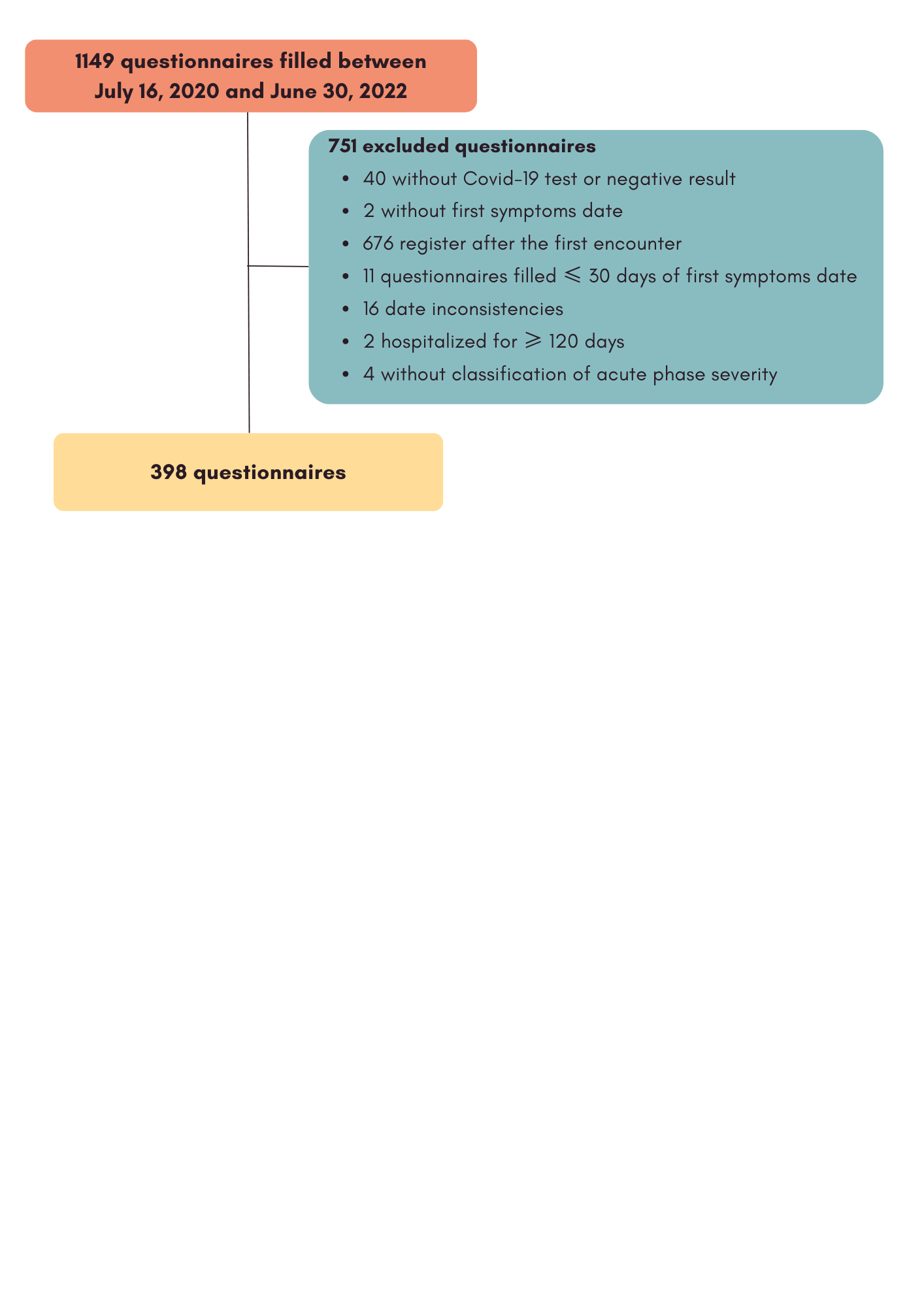


# **Figure S2.** Selection of patients followed up by the post-COVID-19 disease unit (PCDU).

# **Table S2.** Comparison between manual and automated reading of medical condition and symptom terms.

| **Medical Conditions** | **Hits** | **Non Hits** | **Total** | **Percentage (hits)** | **p-value** | **X-squared** |
| --- | --- | --- | --- | --- | --- | --- |
| **Hyperthyroidism** | 396 | 2 | 398 | 0,99 | < 0,001 | 297.74 |
| **Obesity** | 395 | 3 | 398 | 0,99 | < 0,001 | 367.49 |
| **Hypertension** | 393 | 5 | 398 | 0,99 | < 0,001 | 773.85 |
| **Hypothyroidism** | 394 | 4 | 398 | 0,99 | < 0,001 | 359.1 |
| **Diabetes mellitus** | 391 | 7 | 398 | 0,98 | < 0,001 | 731.25 |
| **Asthma** | 390 | 8 | 398 | 0,98 | < 0,001 | 603.58 |
| **Pneumonia** | 390 | 8 | 398 | 0,98 | < 0,001 | 327.45 |
| **COPD** | 387 | 11 | 398 | 0,97 | < 0,001 | 248.03 |
| **Smoking** | 371 | 27 | 398 | 0,93 | < 0,001 | 425.01 |
| **Former smoker** | 369 | 29 | 398 | 0,93 | < 0,001 | 275.68 |
| **Symptoms** | **Hits** | **Non Hits** | **Total** | **Percentage (hits)** | **P-value** | **X-squared** |
| **Cough** | 177 | 18 | 195 | 0,91 | < 0,001 | 290.27 |
| **Fatigue** | 169 | 26 | 195 | 0,87 | < 0,001 | 202.95 |
| **Myalgia** | 176 | 19 | 195 | 0,90 | < 0,001 | 125.66 |
| **Headache** | 186 | 9 | 195 | 0,95 | < 0,001 | 231.72 |

# **TABLE S3**. Odds Ratio with 95% confidence interval from study population with and without long Covid reported symptoms.

| **Characteristic** | **OR***^1^* | **95% CI***^1^* | **p-value** |
| --- | --- | --- | --- |
| **Number of Medical Conditions** |  |  |  |
| 0 | — | — |  |
| 1 | 1.24 | 0.68, 2.25 | 0.5 |
| 2 | 1.34 | 0.72, 2.52 | 0.4 |
| 3+ | 1.67 | 0.85, 3.32 | 0.14 |
| **Gender** |  |  |  |
| Male | — | — |  |
| Female | 1.03 | 0.65, 1.63 | >0.9 |
| **Ethnicity** |  |  |  |
| White | — | — |  |
| Black | 1.22 | 0.59, 2.69 | 0.6 |
| Mixed brown | 0.65 | 0.40, 1.08 | 0.10 |
| Others | 1.49 | 0.36, 10.1 | 0.6 |
| **Age Range** |  |  |  |
| 19-39 | — | — |  |
| 40-59 | 0.66 | 0.31, 1.34 | 0.3 |
| 60-79 | 1.06 | 0.47, 2.29 | 0.9 |
| 80+ | 0.88 | 0.27, 3.02 | 0.8 |
|  |  |  | *^1^* OR = Odds Ratio, CI = Confidence Interval |
